# Supplementary material for: Four dimensions characterize attributions from faces using a representative set of English trait words
Source: Nat Commun. 2021 Aug 27;12:5168. doi: 10.1038/s41467-021-25500-y (PMC8397784; doi:10.1038/s41467-021-25500-y)
Supplement: Supplementary file 1 — Supplementary Information [file 41467_2021_25500_MOESM1_ESM.pdf]

Supplementary Information

Lin et al.

Supplementary Figures

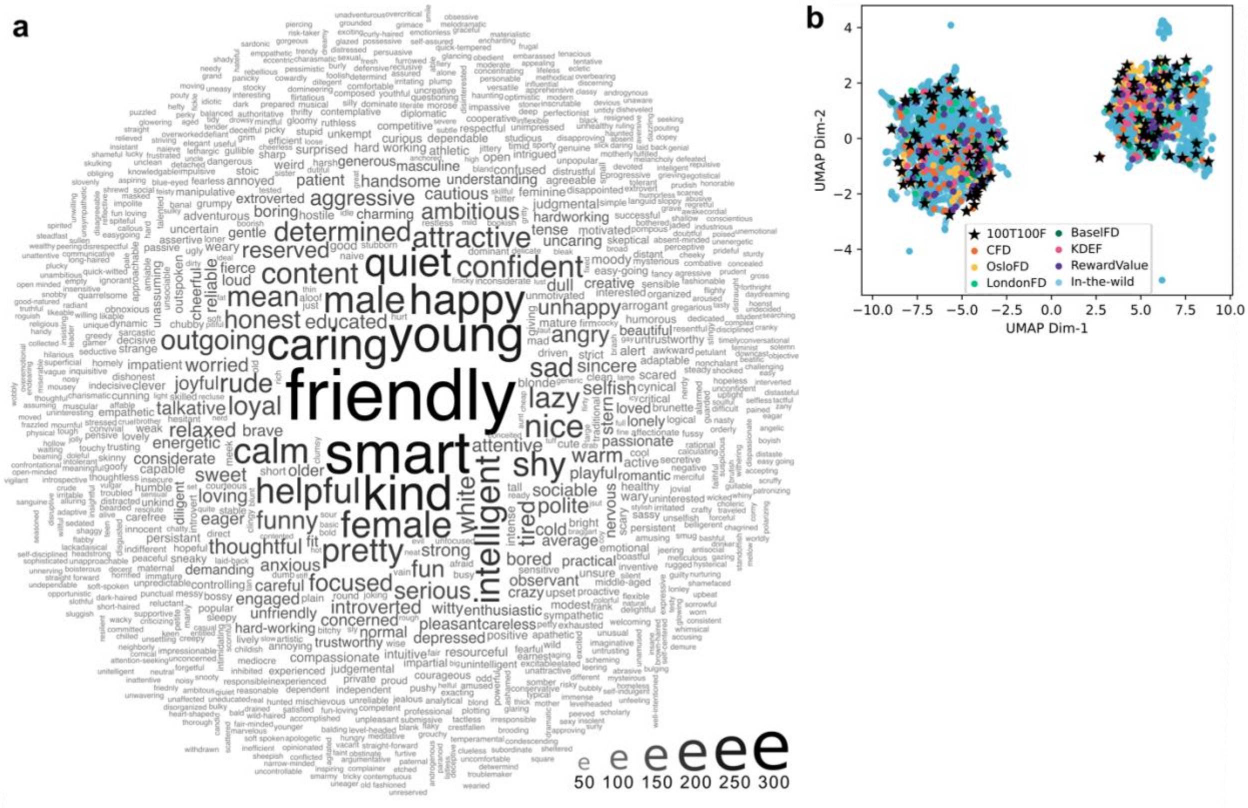

Supplementary Figure 1: Verifying the representativeness of the 100 selected traits and face images.

a, Word cloud of the 973 freely generated descriptions of the 100 selected face images during spontaneous face judgments (see Methods). All words that appeared at least twice are shown in the figure (words that appeared only once were excluded, as they were comprised mainly of misspelled words or words not included in the FastText vocabulary[1]). The scale indicates the frequency the word was mentioned (ranging from 2 to 306 times). **b,** Uniform Manifold Approximation and Projection (UMAP[2]) of the 100 selected face images (stars), frontal, neutral, white face images from multiple databases (dots that are not light blue, N = 632), and ambient

face images in real world contexts[3,4] (light blue dots; with various angles, gazes, facial expressions, lighting, backgrounds, etc.; N = 4744). All faces were represented by the 128 computationally extracted features used by a state-of-the-art neural network for facial recognition[5].

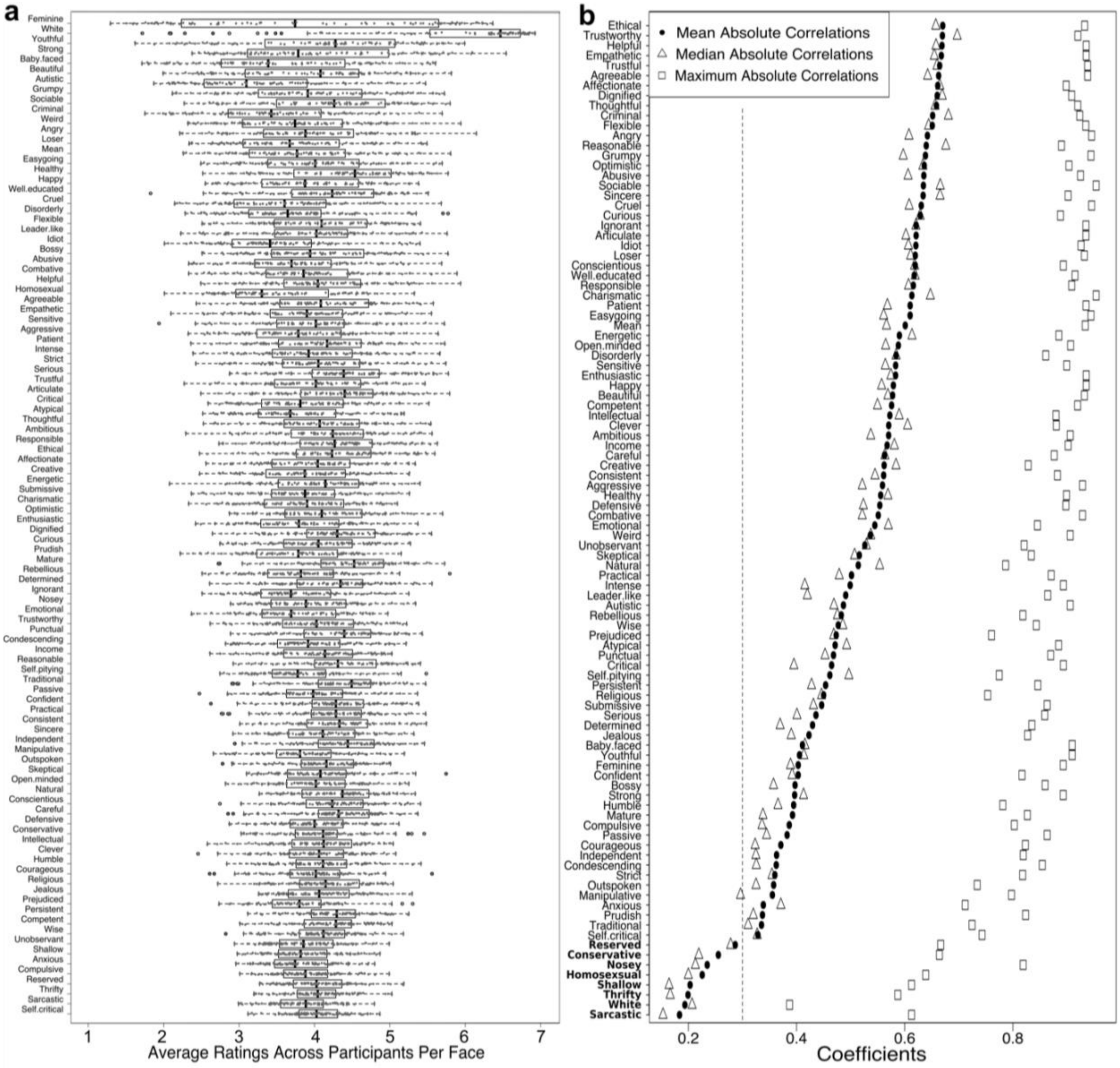

Supplementary Figure 2: Variance and factorizability of ratings across 100 traits.

a, Distribution of aggregate-level trait ratings. Each row plots the average ratings across participants for the 100 faces on a trait (grey dots), with the median (line in the box), the first quartile (left edge of the box), the third quartile (right edge of the box), and outliers that are more extreme than 3/2 times of the quartiles (open dots). We supplemented the 94 traits selected from the literature with additional words for which we believed there was no equivalent in the initial list but would reflect vocabulary used to describe first impressions, including words that describe

sexual orientation (homosexual), traits associated with a neurodevelopmental disorder (autistic), perceived demographics (high-income, well-educated), and words that are considered derogatory terms for an individual's intellectual or social ability (idiot, loser). **b,** Factorability of aggregate-level trait ratings. Each row plots the mean (dot), median (triangle), and maximum (square) absolute correlations a trait has with all the other 99 traits (across faces, averaged over participants). The vertical dashed line indicates $r = 0.30$, which describes an inflection point in the curve of mean absolute correlations. The eight traits at the bottom (in bold) were excluded from EFA because of their low average correlations with all other traits (i.e. low factorizability); including these eight traits did not change the dimensions we eventually found. Source data are provided as a Source Data file.

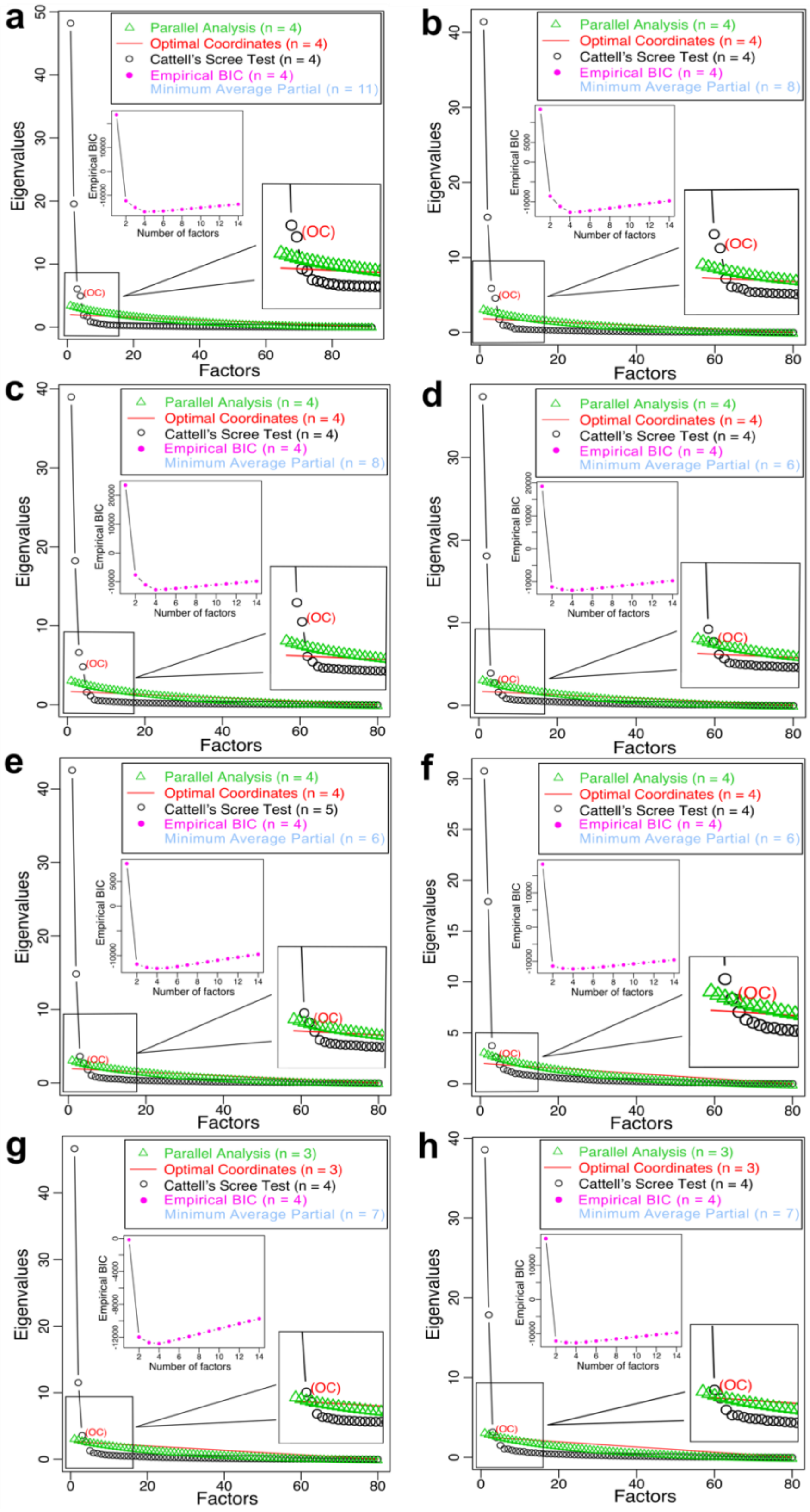

Supplementary Figure 3: Scree plots of data across eight samples.

a, Study 1 sample. **b-h,** Study 2 samples from North America (b), Latvia (c), Peru (d), the Philippines (e), India (f), Kenya (g), and Gaza (h). Circles plot the eigenvalues (the fraction of total common variance in the data as explained by each factor) of the original data across factors, ordered from the largest to the smallest. Triangles plot the 95th percentile of the eigenvalues of the simulated data from parallel analysis. The optimal number of factors to retain as recommended by each of the five methods is shown. Parallel analysis retains factors with eigenvalues (circles) greater than those from the simulated data (triangles) from 5,000 Monte Carlo simulations (see the close-up image for a clearer comparison). Cattell's scree test retains factors to the left of the point from which the plotted ordered eigenvalues could be approximated with a straight line (i.e., "above the elbow"). The optimal coordinates index provides a non-graphical solution to Cattell's scree test based on linear extrapolation. Empirical Bayesian information criterion (eBIC) retains factors that minimize the overall discrepancy between the population's and the model's predicted covariance matrices while penalizing model complexity (purple dots in inset graphs). Velicer's minimum average partial (MAP) test is "most appropriate when component analysis is employed as an alternative to, or a first-stage solution for, factor analysis"[8]. It is also included in our present study due to its popularity. MAP retains components by partialing those that resulted in the lowest average squared partial correlation. The MAP test gave variable numbers of components greater than 4; it is not plotted but the results are numerically provided in the legend inset. Source data are provided as a Source Data file.

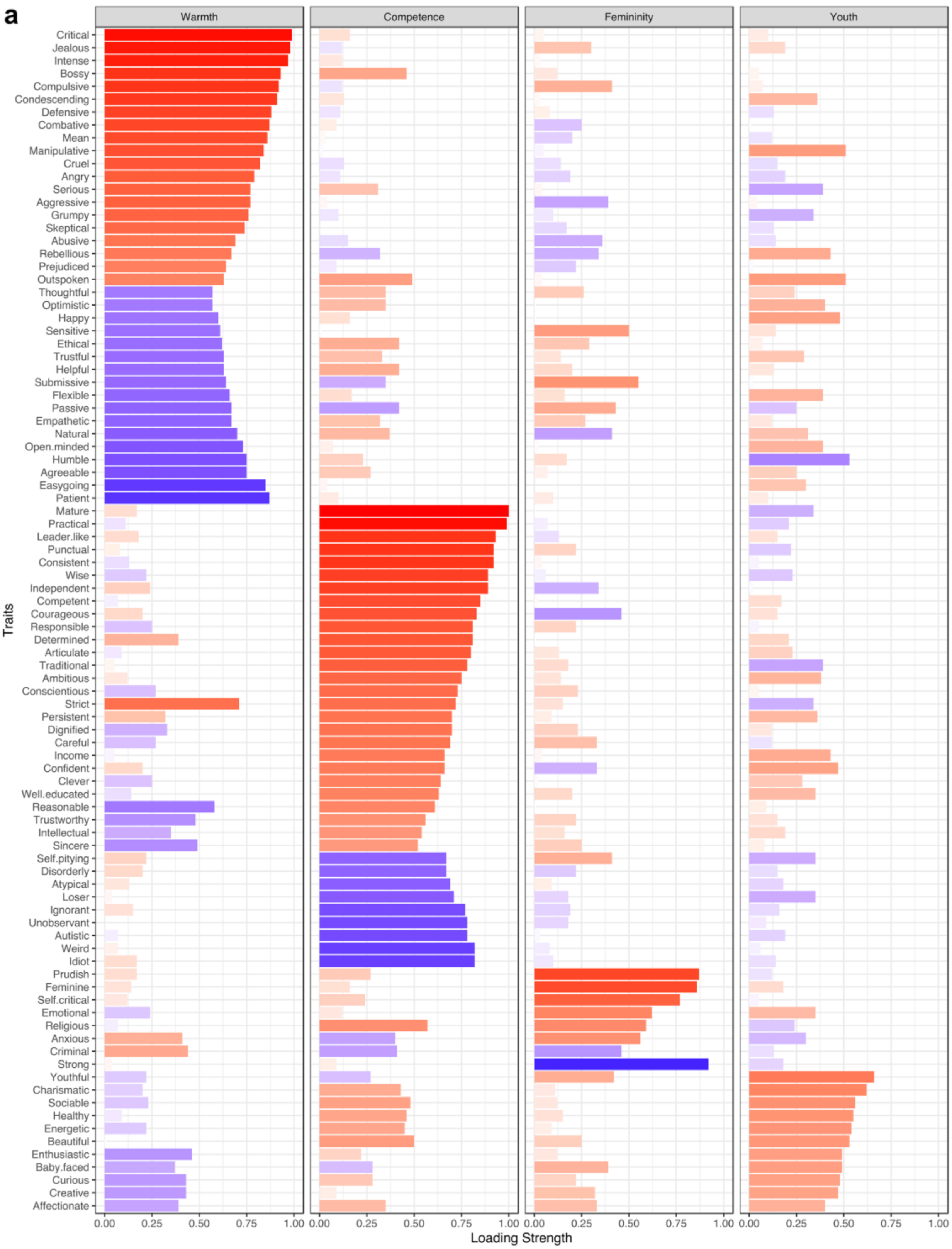
a
Warmth
Competence
Femininity
Youth
Traits
Critical
Jealous
Intense
Bossy
Compulsive
Condescending
Defensive
Combative
Mean
Manipulative
Cruel
Angry
Serious
Aggressive
Grumpy
Skeptical
Abusive
Rebellious
Prejudiced
Outspoken
Thoughtful
Optimistic
Happy
Sensitive
Ethical
Trustful
Helpful
Submissive
Flexible
Passive
Empathetic
Natural
Open.minded
Humble
Agreeable
Easygoing
Patient
Mature
Practical
Leader.like
Punctual
Consistent
Wise
Independent
Competent
Courageous
Responsible
Determined
Articulate
Traditional
Ambitious
Conscientious
Strict
Persistent
Dignified
Careful
Income
Confident
Clever
Well.educated
Reasonable
Trustworthy
Intellectual
Sincere
Self.pitying
Disorderly
Atypical
Loser
Ignorant
Unobservant
Autistic
Weird
Idiot
Prudish
Feminine
Self.critical
Emotional
Religious
Anxious
Criminal
Strong
Youthful
Charismatic
Sociable
Healthy
Energetic
Beautiful
Enthusiastic
Baby.faced
Curious
Creative
Affectionate
0.00
0.25
0.50
0.75
1.00
Loading Strength

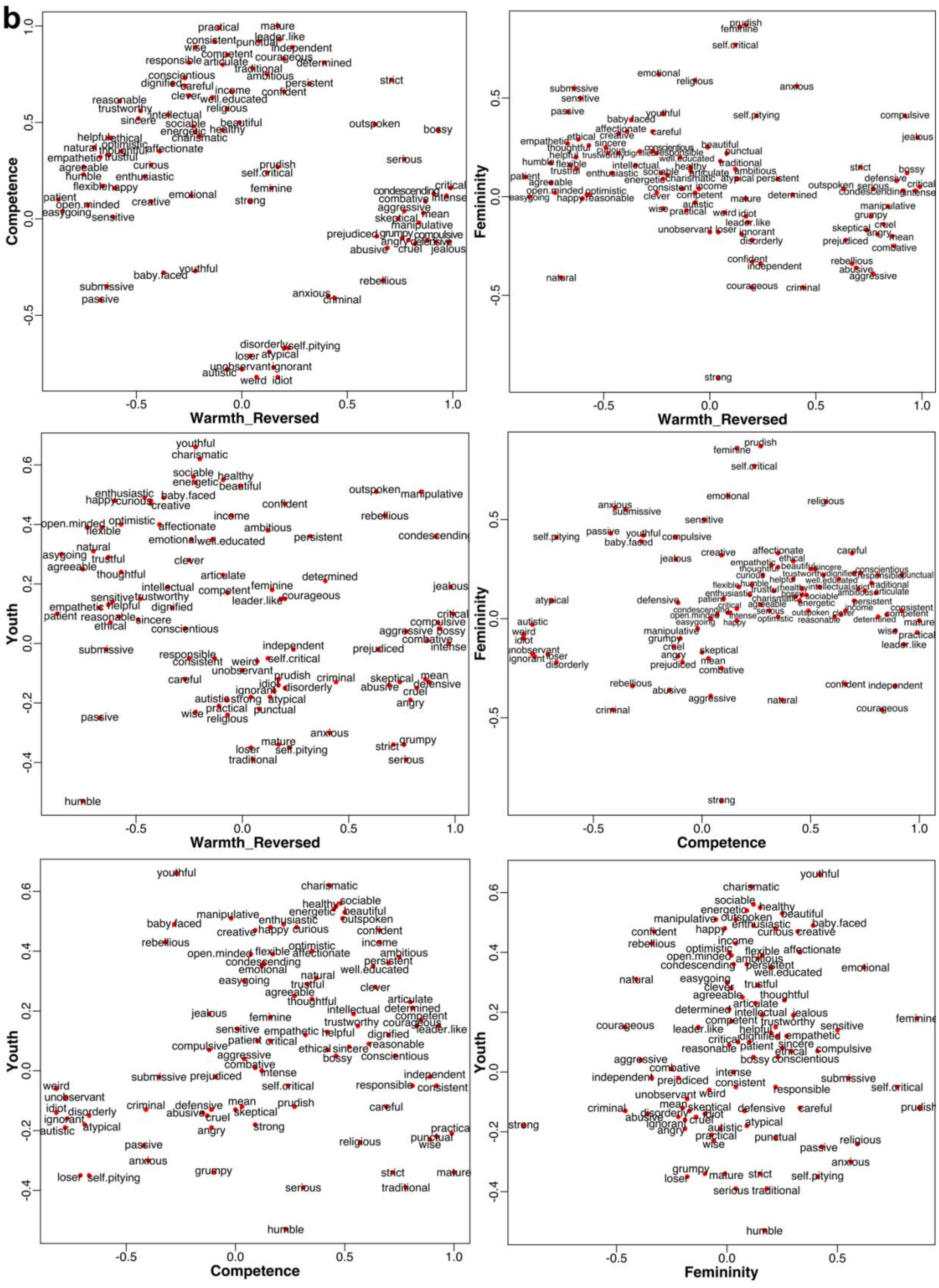
b
Competence
Warmth_Reversed
Femininity
Warmth_Reversed
Youth
Warmth_Reversed
Femininity
Competence
Youth
Competence
Youth
Femininity

1

Supplementary Figure 4: Four dimensions from EFA in Study 1.

a, Factor loadings of trait ratings on the four dimensions from EFA. Each column plots the strength of the factor loadings (x-axis, absolute value) across traits (y-axis). Color indicates the sign of the loading (red for positive and blue for negative); more saturated colors for higher absolute values. **b,** Distributions of the 92 traits along each pair of dimensions based on their factor loadings on the four dimensions. Source data are provided as a Source Data file.

a

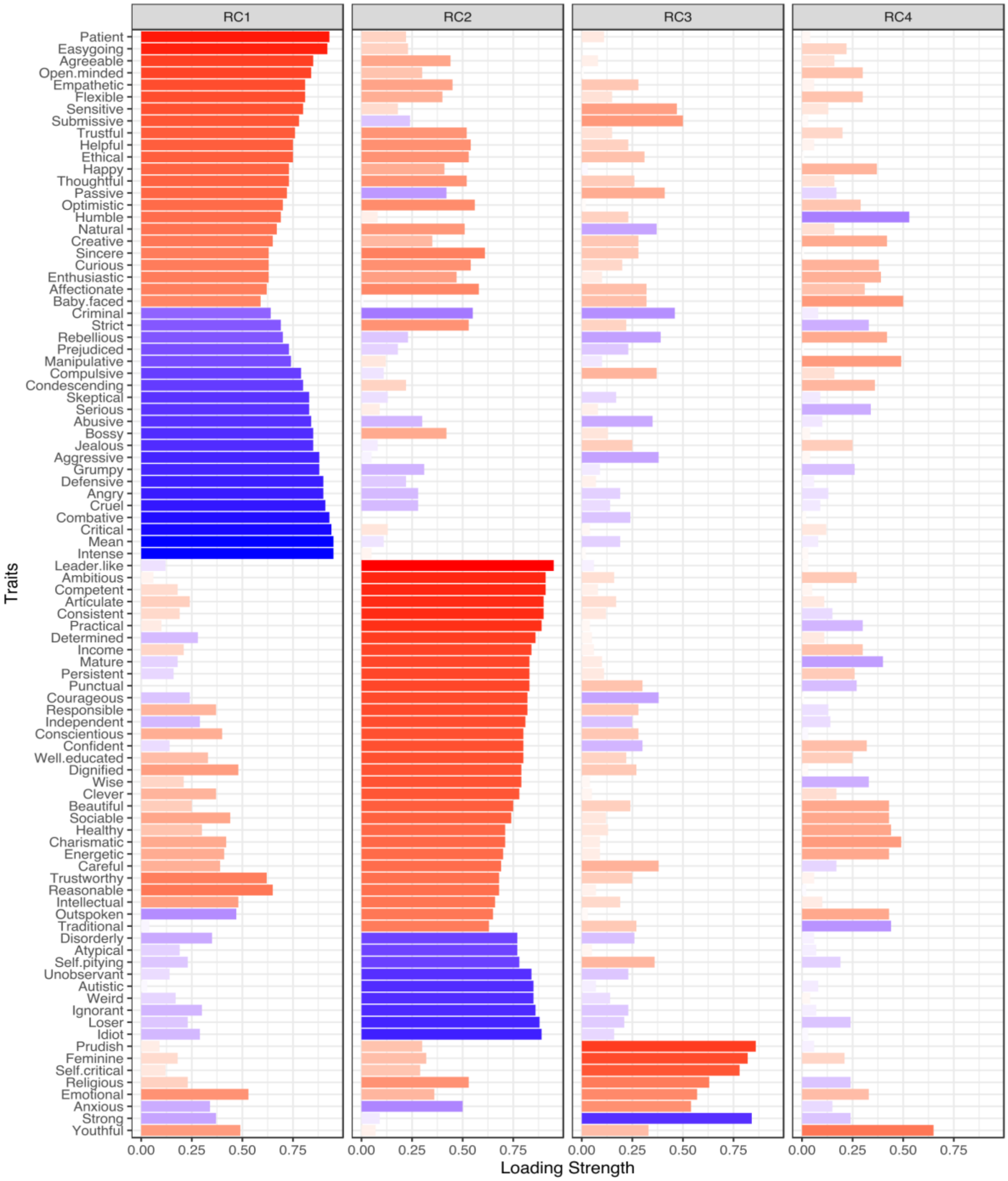
RC1
RC2
RC3
RC4
Traits
Patient
Easygoing
Agreeable
Open.minded
Empathetic
Flexible
Sensitive
Submissive
Trustful
Helpful
Ethical
Happy
Thoughtful
Passive
Optimistic
Humble
Natural
Creative
Sincere
Curious
Enthusiastic
Affectionate
Baby.faced
Criminal
Strict
Rebellious
Prejudiced
Manipulative
Compulsive
Condescending
Skeptical
Serious
Abusive
Bossy
Jealous
Aggressive
Grumpy
Defensive
Angry
Cruel
Combative
Critical
Mean
Intense
Leader.like
Ambitious
Competent
Articulate
Consistent
Practical
Determined
Income
Mature
Persistent
Punctual
Courageous
Responsible
Independent
Conscientious
Confident
Well.educated
Dignified
Wise
Clever
Beautiful
Sociable
Healthy
Charismatic
Energetic
Careful
Trustworthy
Reasonable
Intellectual
Outspoken
Traditional
Disorderly
Atypical
Self.pitying
Unobservant
Autistic
Weird
Ignorant
Loser
Idiot
Prudish
Feminine
Self.critical
Religious
Emotional
Anxious
Strong
Youthful
0.00
0.25
0.50
0.75
Loading Strength

b

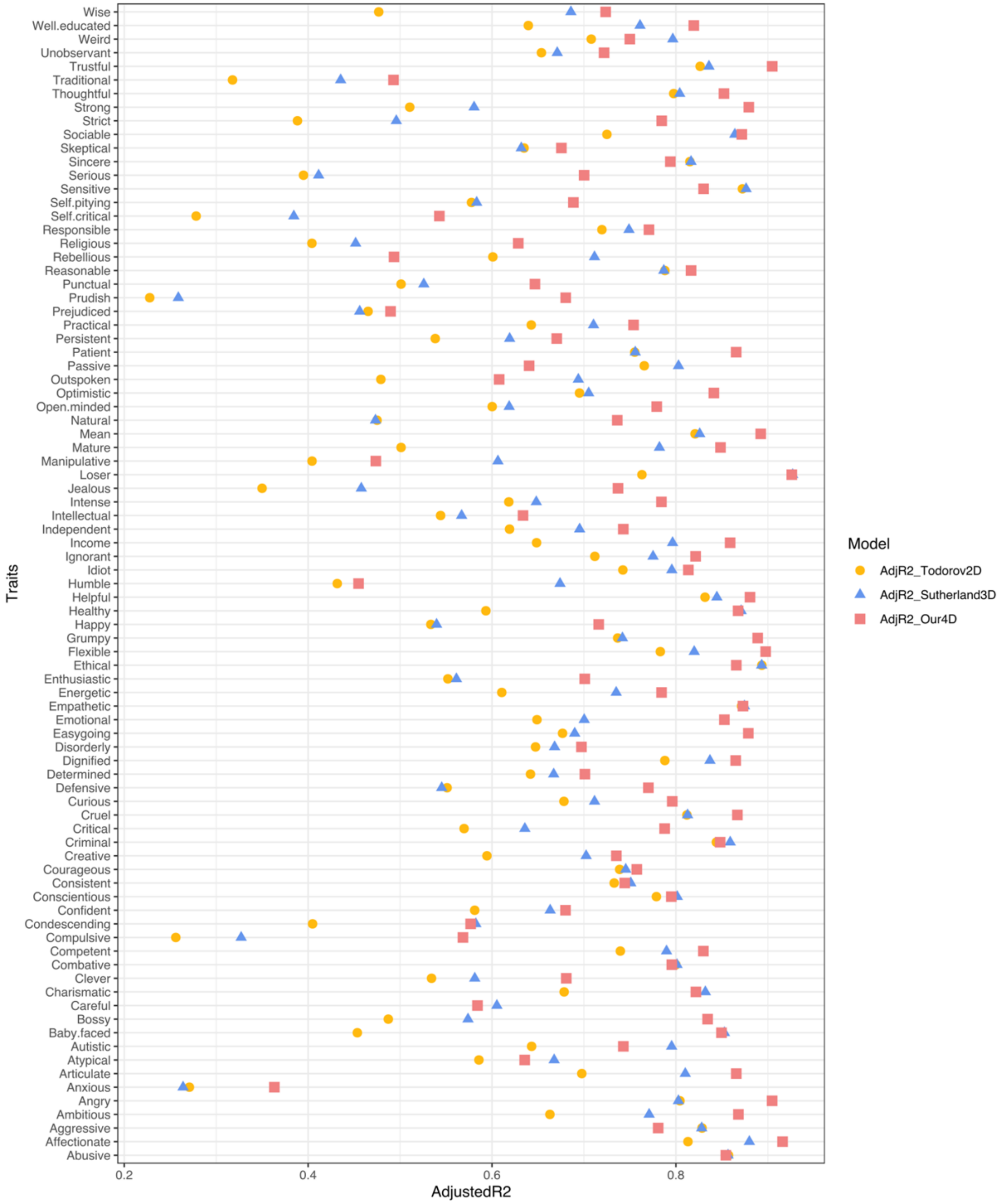

Supplementary Figure 5: Comparison with existing dimensional frameworks.

a, Four dimensions from PCA in Study 1. Columns plot the strength of the loadings (x-axis, absolute value) on the first four varimax rotated principal components across all 92 traits (y-axis). Colors indicate the sign of the loading (red for positive and blue for negative); more saturated colors for higher absolute values. The first four principal components without rotation accounted for 52%, 21%, 7%, and 5% of the variance in our data, 86% in total; the fifth accounted for 2%. **b,** Predicting trait judgments using different dimensional frameworks. Regressors were linear combinations of the traits that showed highest loadings in each dimensional framework (two traits for each dimension because there were only two traits that loaded highest on one of the 3D-framework's dimensions[9]; for example, for our 4D-framework, the model consisted of eight regressors). Each row indicates three different models that regressed the ratings of a targeted trait (row name; which was not one of the regressors) on the three different sets of regressors from the three frameworks, and plots the adjusted R-squared. Source data are provided as a Source Data file.

a

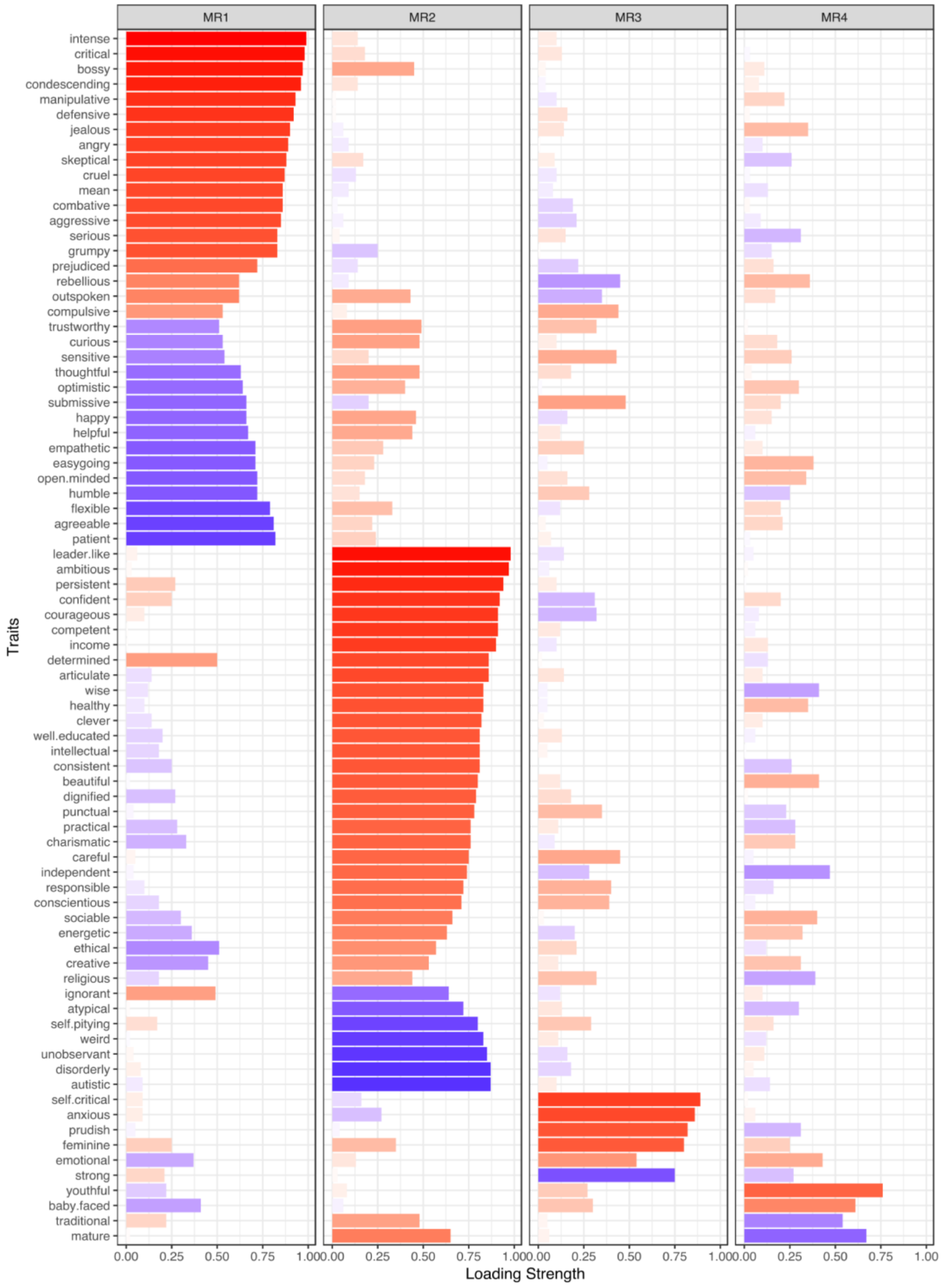
MR1
MR2
MR3
MR4
Traits
intense
critical
bossy
condescending
manipulative
defensive
jealous
angry
skeptical
cruel
mean
combative
aggressive
serious
grumpy
prejudiced
rebellious
outspoken
compulsive
trustworthy
curious
sensitive
thoughtful
optimistic
submissive
happy
helpful
empathetic
easygoing
open.minded
humble
flexible
agreeable
patient
leader.like
ambitious
persistent
confident
courageous
competent
income
determined
articulate
wise
healthy
clever
well.educated
intellectual
consistent
beautiful
dignified
punctual
practical
charismatic
careful
independent
responsible
conscientious
sociable
energetic
ethical
creative
religious
ignorant
atypical
self.pitying
weird
unobservant
disorderly
autistic
self.critical
anxious
prudish
feminine
emotional
strong
youthful
baby.faced
traditional
mature
0.00
0.25
0.50
0.75
1.00
Loading Strength

b

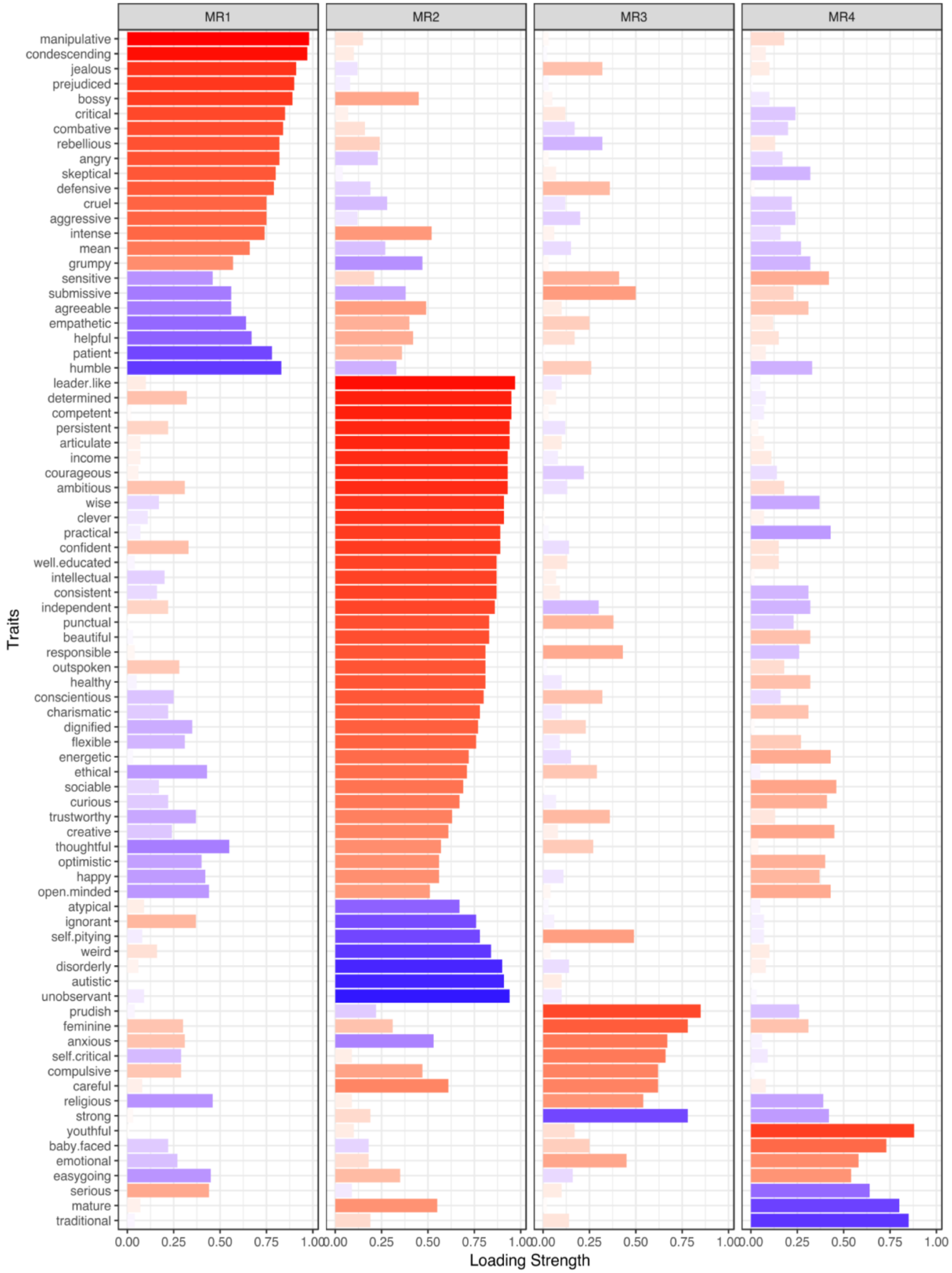
MR1
MR2
MR3
MR4
Traits
manipulative
condescending
jealous
prejudiced
bossy
critical
combative
rebellious
angry
skeptical
defensive
cruel
aggressive
intense
mean
grumpy
sensitive
submissive
agreeable
empathetic
helpful
patient
humble
leader.like
determined
competent
persistent
articulate
income
courageous
ambitious
wise
clever
practical
confident
well.educated
intellectual
consistent
independent
punctual
beautiful
responsible
outspoken
healthy
conscientious
charismatic
dignified
flexible
energetic
ethical
sociable
curious
trustworthy
creative
thoughtful
optimistic
happy
open.minded
atypical
ignorant
self.pitying
weird
disorderly
autistic
unobservant
prudish
feminine
anxious
self.critical
compulsive
careful
religious
strong
youthful
baby.faced
emotional
easygoing
serious
mature
traditional
0.00
0.25
0.50
0.75
1.00
Loading Strength

c

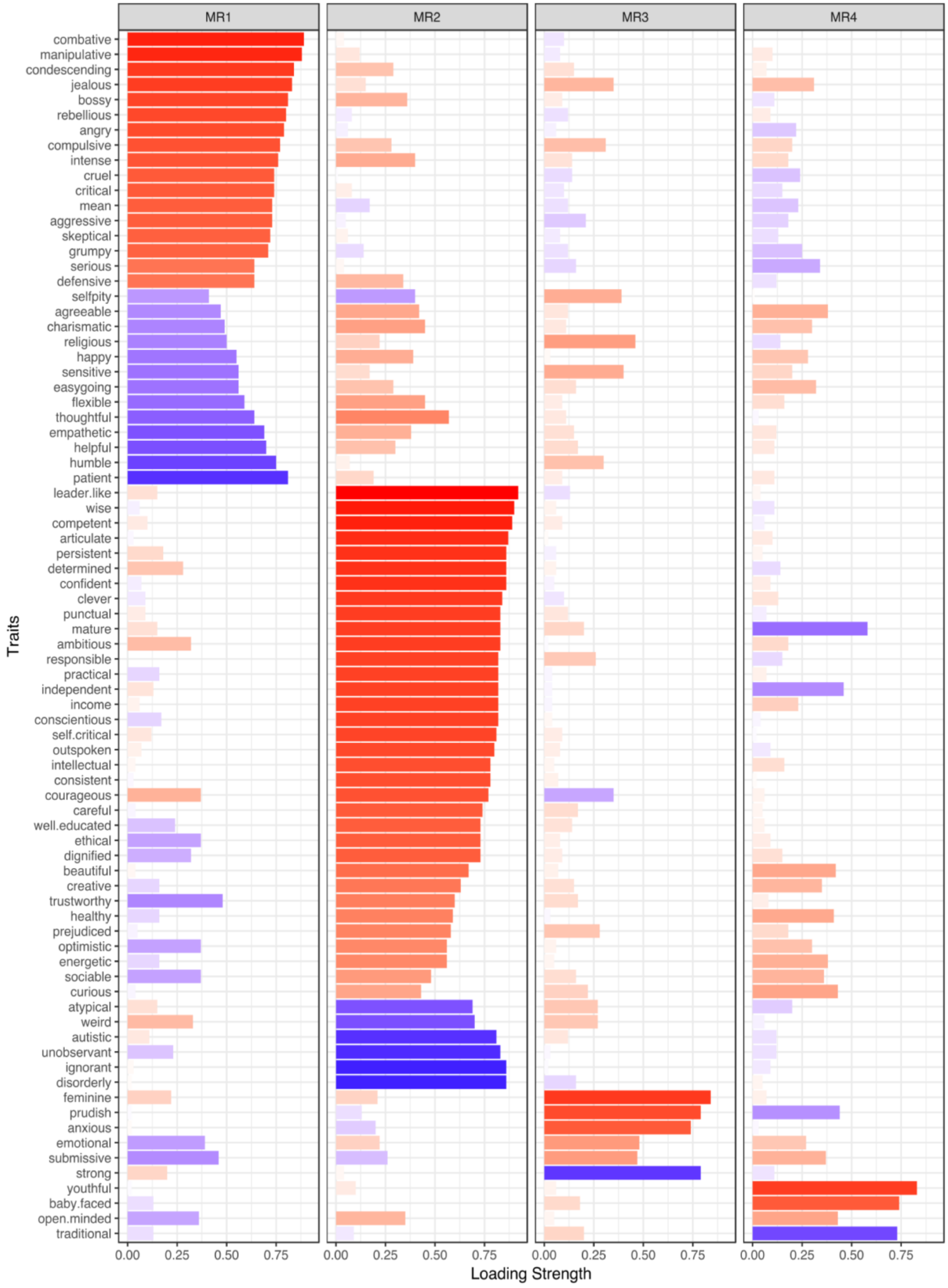
MR1
MR2
MR3
MR4
combative
manipulative
condescending
jealous
bossy
rebellious
angry
compulsive
intense
cruel
critical
mean
aggressive
skeptical
grumpy
serious
defensive
selfpity
agreeable
charismatic
religious
happy
sensitive
easygoing
flexible
thoughtful
empathetic
helpful
humble
patient
leader.like
wise
competent
articulate
persistent
determined
confident
clever
punctual
mature
ambitious
responsible
practical
independent
income
conscientious
self.critical
outspoken
intellectual
consistent
courageous
careful
well.educated
ethical
dignified
beautiful
creative
trustworthy
healthy
prejudiced
optimistic
energetic
sociable
curious
atypical
weird
autistic
unobservant
ignorant
disorderly
feminine
prudish
anxious
emotional
submissive
strong
youthful
baby.faced
open.minded
traditional
Traits
0.00
0.25
0.50
0.75
Loading Strength

d

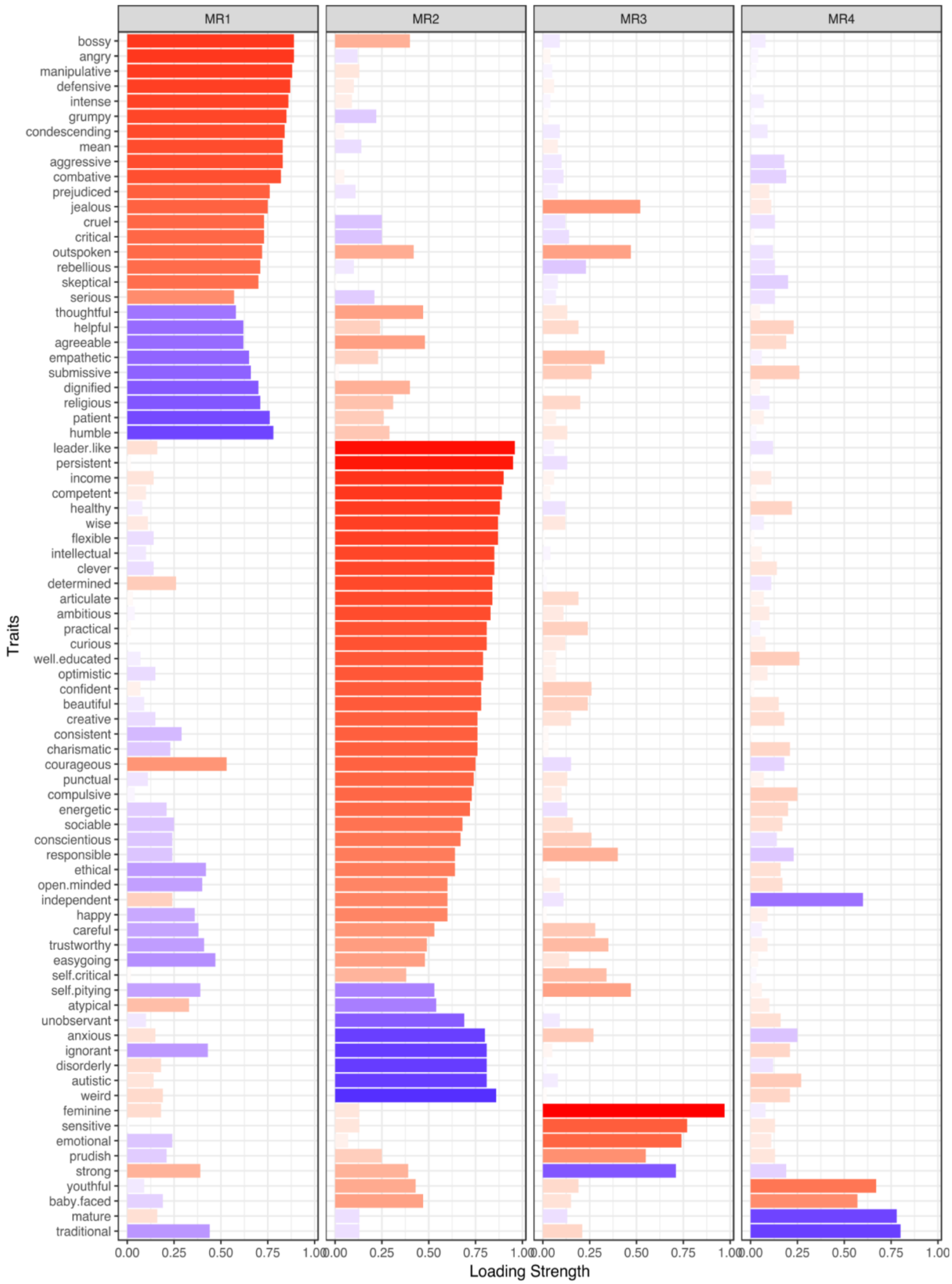
MR1
MR2
MR3
MR4
Traits
bossy
angry
manipulative
defensive
intense
grumpy
condescending
mean
aggressive
combative
prejudiced
jealous
cruel
critical
outspoken
rebellious
skeptical
serious
thoughtful
helpful
agreeable
empathetic
submissive
dignified
religious
patient
humble
leader.like
persistent
income
competent
healthy
wise
flexible
intellectual
clever
determined
articulate
ambitious
practical
curious
well.educated
optimistic
confident
beautiful
creative
consistent
charismatic
courageous
punctual
compulsive
energetic
sociable
conscientious
responsible
ethical
open.minded
independent
happy
careful
trustworthy
easygoing
self.critical
self.pitying
atypical
unobservant
anxious
ignorant
disorderly
autistic
weird
feminine
sensitive
emotional
prudish
strong
youthful
baby.faced
mature
traditional
0.00 0.25 0.50 0.75 1.00
Loading Strength

e

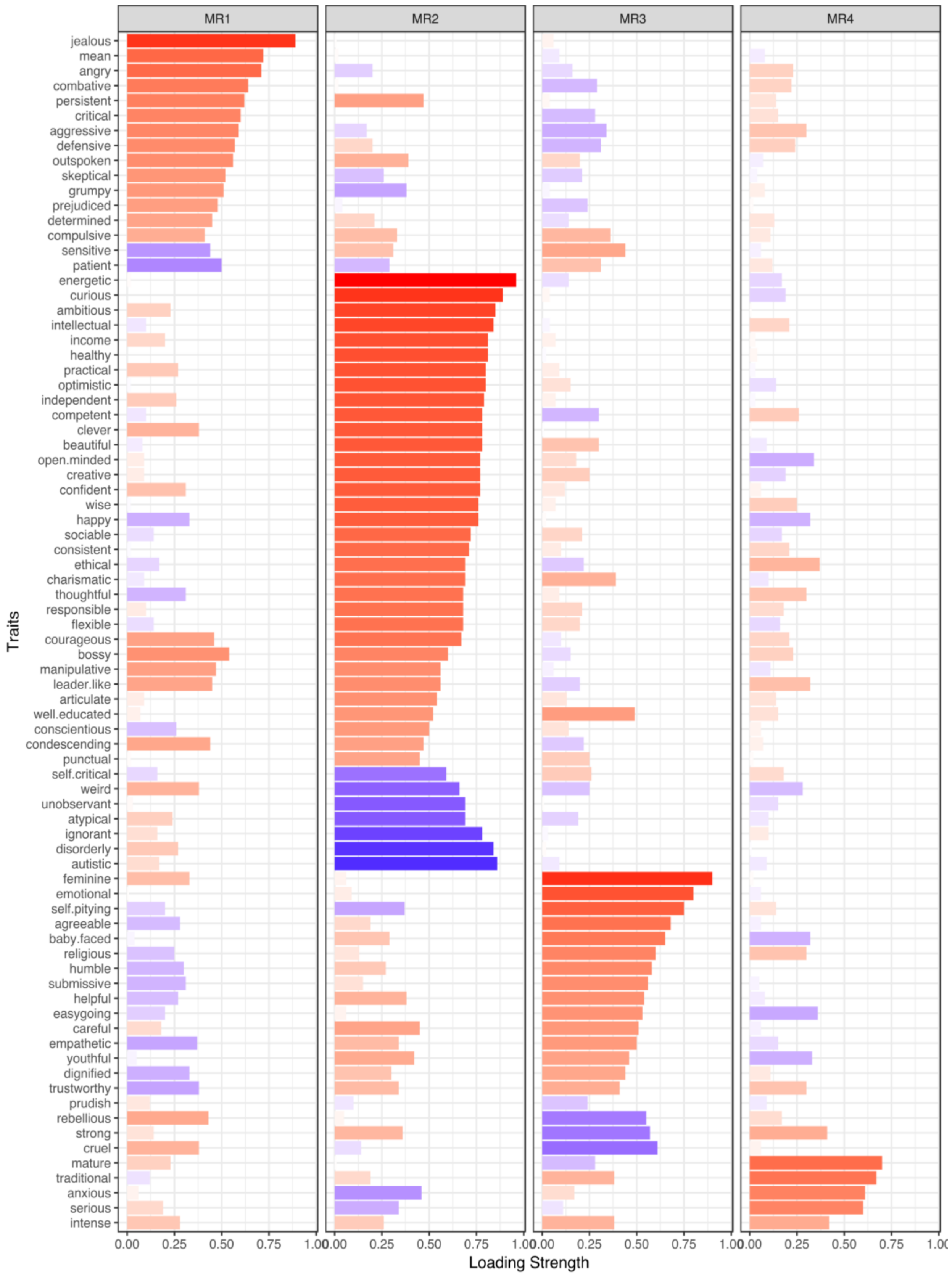

MR1
MR2
MR3
MR4
Traits
jealous
mean
angry
combative
persistent
critical
aggressive
defensive
outspoken
skeptical
grumpy
prejudiced
determined
compulsive
sensitive
patient
energetic
curious
ambitious
intellectual
income
healthy
practical
optimistic
independent
competent
clever
beautiful
open.minded
creative
confident
wise
happy
sociable
consistent
ethical
charismatic
thoughtful
responsible
flexible
courageous
bossy
manipulative
leader.like
articulate
well.educated
conscientious
condescending
punctual
self.critical
weird
unobservant
atypical
ignorant
disorderly
autistic
feminine
emotional
self.pitying
agreeable
baby.faced
religious
humble
submissive
helpful
easygoing
careful
empathetic
youthful
dignified
trustworthy
prudish
rebellious
strong
cruel
mature
traditional
anxious
serious
intense
0.00
0.25
0.50
0.75
1.00
Loading Strength

f

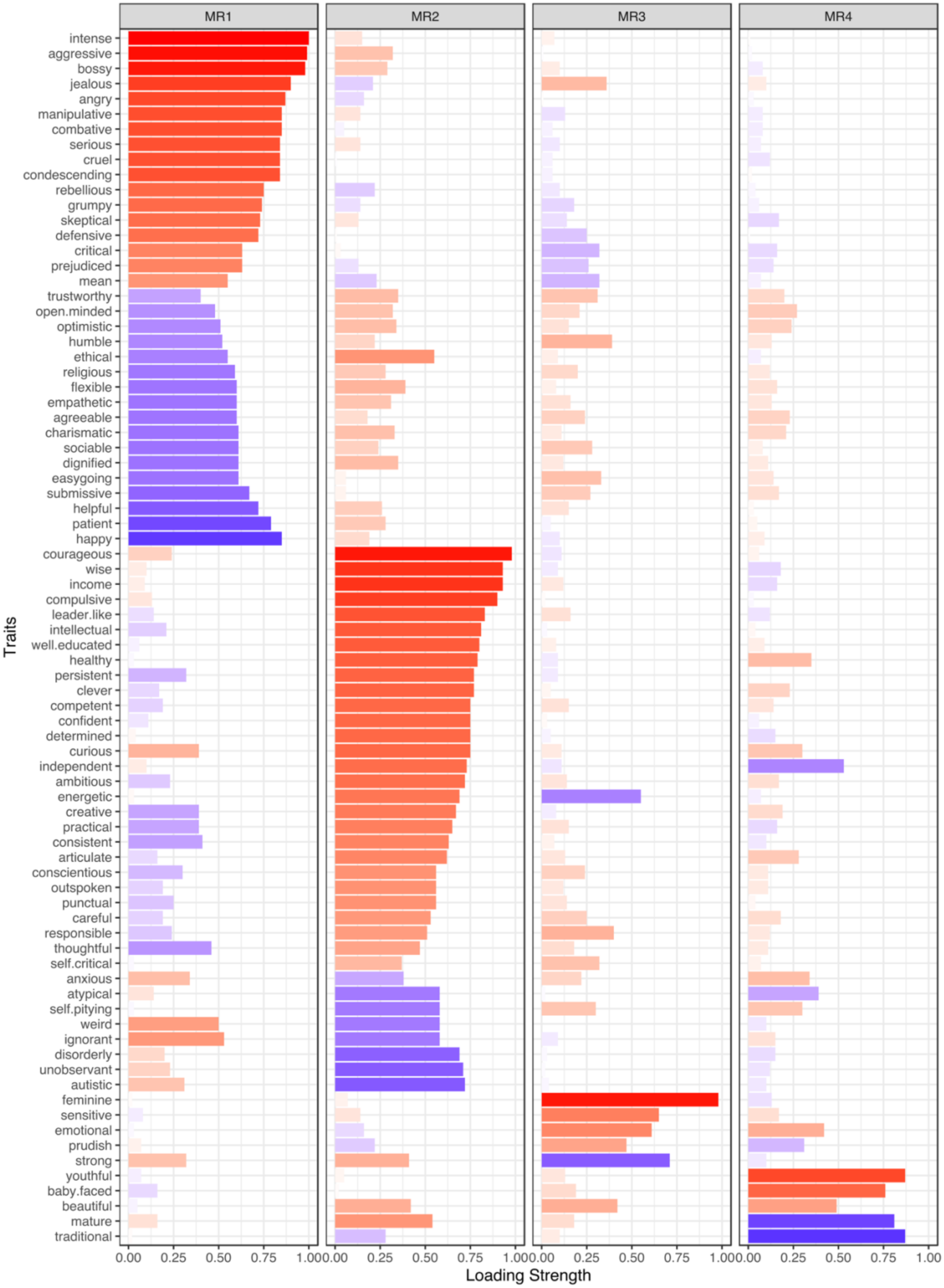
MR1
MR2
MR3
MR4
Traits
intense
aggressive
bossy
jealous
angry
manipulative
combative
serious
cruel
condescending
rebellious
grumpy
skeptical
defensive
critical
prejudiced
mean
trustworthy
open.minded
optimistic
humble
ethical
religious
flexible
empathetic
agreeable
charismatic
sociable
dignified
easygoing
submissive
helpful
patient
happy
courageous
wise
income
compulsive
leader.like
intellectual
well.educated
healthy
persistent
clever
competent
confident
determined
curious
independent
ambitious
energetic
creative
practical
consistent
articulate
conscientious
outspoken
punctual
careful
responsible
thoughtful
self.critical
anxious
atypical
self.pitying
weird
ignorant
disorderly
unobservant
autistic
feminine
sensitive
emotional
prudish
strong
youthful
baby.faced
beautiful
mature
traditional
0.00
0.25
0.50
0.75
1.00
Loading Strength

g

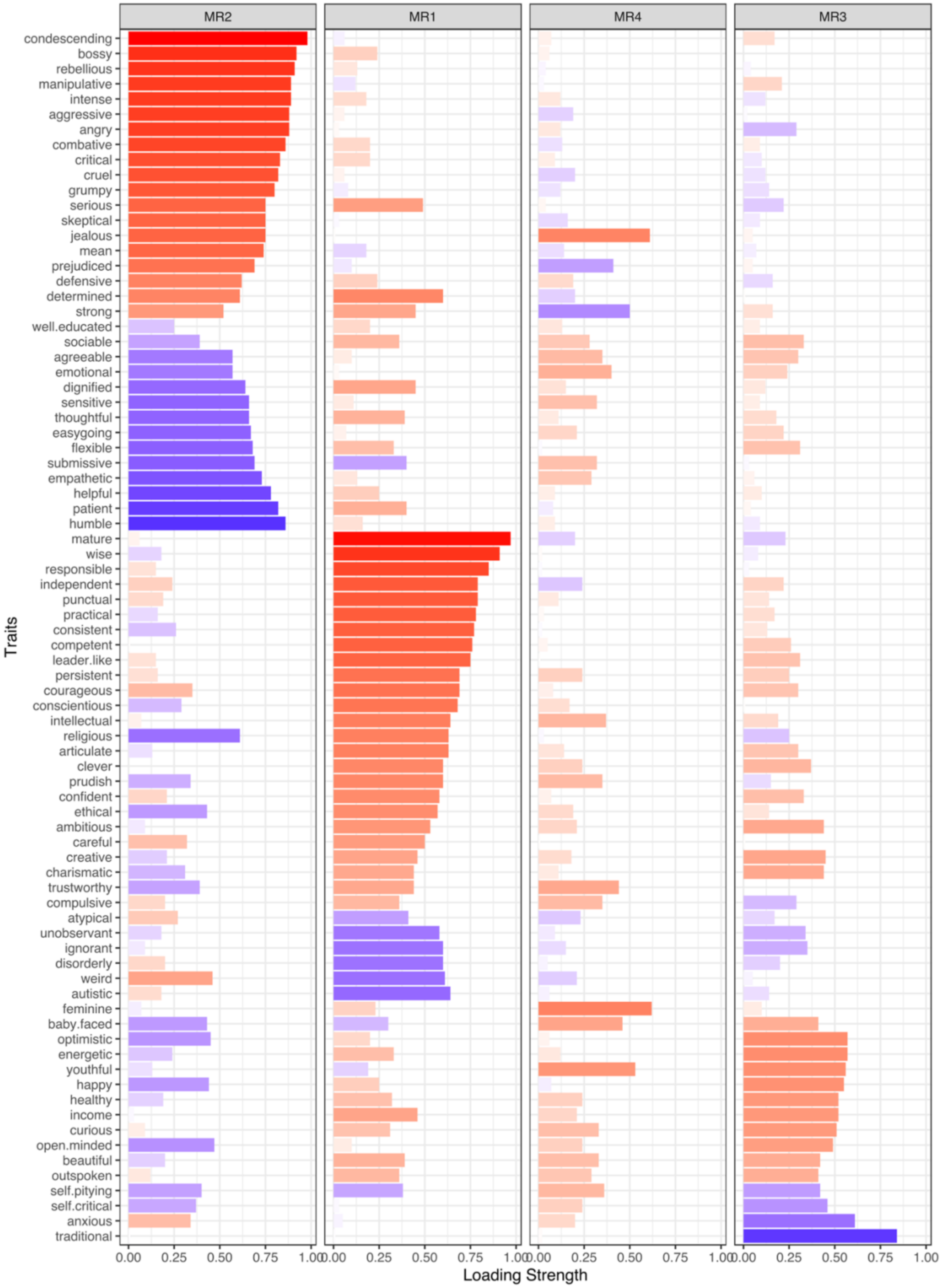
MR2
MR1
MR4
MR3
Traits
condescending
bossy
rebellious
manipulative
intense
aggressive
angry
combative
critical
cruel
grumpy
serious
skeptical
jealous
mean
prejudiced
defensive
determined
strong
well.educated
sociable
agreeable
emotional
dignified
sensitive
thoughtful
easygoing
flexible
submissive
empathetic
helpful
patient
humble
mature
wise
responsible
independent
punctual
practical
consistent
competent
leader.like
persistent
courageous
conscientious
intellectual
religious
articulate
clever
prudish
confident
ethical
ambitious
careful
creative
charismatic
trustworthy
compulsive
atypical
unobservant
ignorant
disorderly
weird
autistic
feminine
baby.faced
optimistic
energetic
youthful
happy
healthy
income
curious
open.minded
beautiful
outspoken
self.pitying
self.critical
anxious
traditional
0.00
0.25
0.50
0.75
1.00
Loading Strength

Supplementary Figure 6: Four factors extracted from aggregated data in Study 2. The seven panels plot results for samples from **a,** North America, **b,** Latvia, **c,** Peru, **d,** the Philippines, **e**, India, **f,** Kenya, and **g,** Gaza. Each column plots the strength of the factor loadings across the 80 traits (20 of the 100 traits were excluded in the present study for low correlations with other traits [see Supplementary Figure 2], ambiguity or similarity in meaning [trustful, natural, passive, reasonable, strict, enthusiastic, affectionate, and sincere], and potential offensiveness in some cultures [idiot, loser, criminal, and abusive]). The color of the bar indicates the sign of the loading (red: positive; blue: negative); the length and saturation of the bar indicates the magnitude of the loading. Source data are provided as a Source Data file.

a1

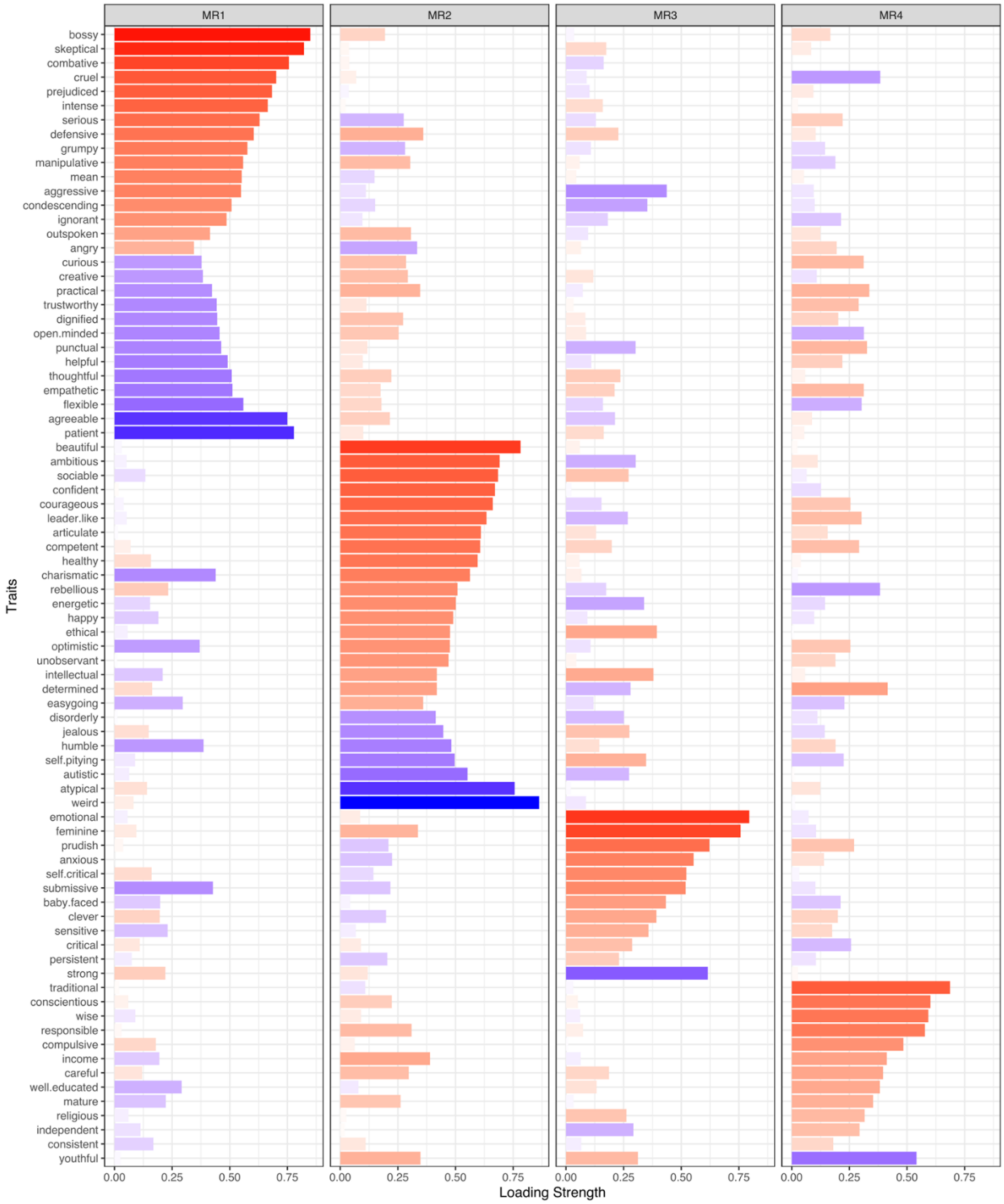
MR1
MR2
MR3
MR4
Traits
bossy
skeptical
combative
cruel
prejudiced
intense
serious
defensive
grumpy
manipulative
mean
aggressive
condescending
ignorant
outspoken
angry
curious
creative
practical
trustworthy
dignified
open.minded
punctual
helpful
thoughtful
empathetic
flexible
agreeable
patient
beautiful
ambitious
sociable
confident
courageous
leader.like
articulate
competent
healthy
charismatic
rebellious
energetic
happy
ethical
optimistic
unobservant
intellectual
determined
easygoing
disorderly
jealous
humble
self.pitying
autistic
atypical
weird
emotional
feminine
prudish
anxious
self.critical
submissive
baby.faced
clever
sensitive
critical
persistent
strong
traditional
conscientious
wise
responsible
compulsive
income
careful
well.educated
mature
religious
independent
consistent
youthful
0.00
0.25
0.50
0.75
Loading Strength

a2

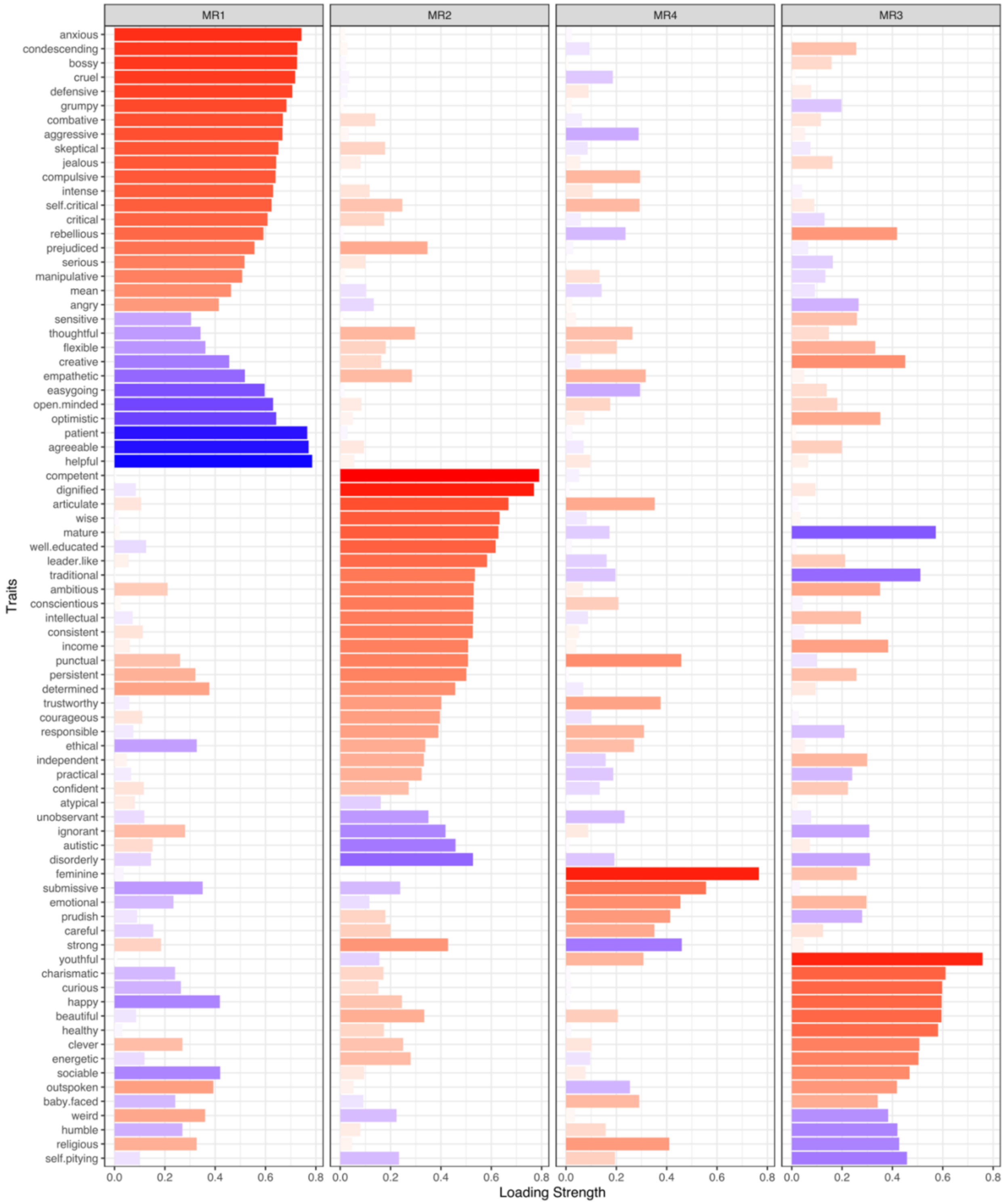
MR1
MR2
MR4
MR3
anxious
condescending
bossy
cruel
defensive
grumpy
combative
aggressive
skeptical
jealous
compulsive
intense
self.critical
critical
rebellious
prejudiced
serious
manipulative
mean
angry
sensitive
thoughtful
flexible
creative
empathetic
easygoing
open.minded
optimistic
patient
agreeable
helpful
competent
dignified
articulate
wise
mature
well.educated
leader.like
traditional
ambitious
conscientious
intellectual
consistent
income
punctual
persistent
determined
trustworthy
courageous
responsible
ethical
independent
practical
confident
atypical
unobservant
ignorant
autistic
disorderly
feminine
submissive
emotional
prudish
careful
strong
youthful
charismatic
curious
happy
beautiful
healthy
clever
energetic
sociable
outspoken
baby.faced
weird
humble
religious
self.pitying
Traits
0.0
0.2
0.4
0.6
0.8
Loading Strength

a3

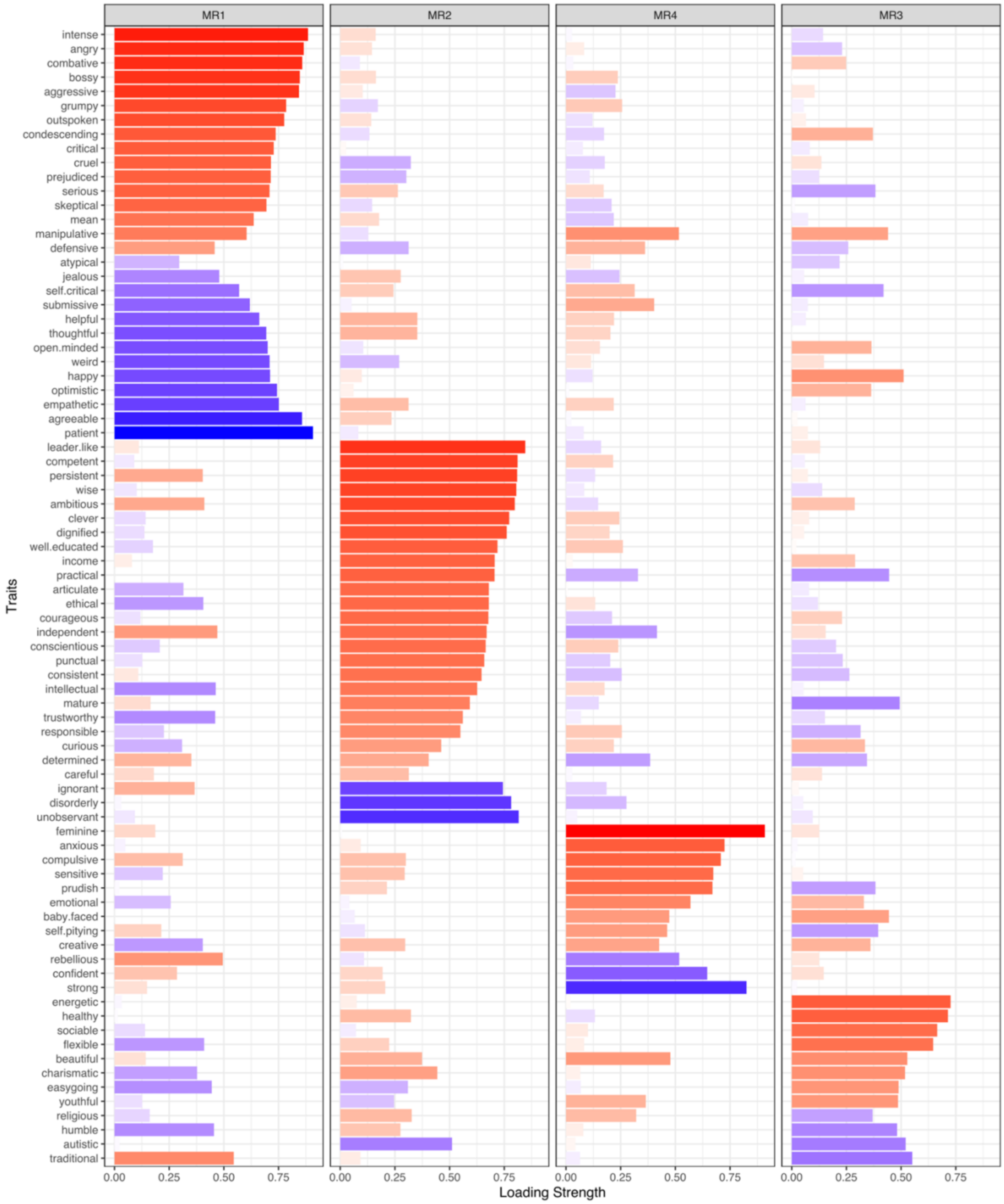
MR1
MR2
MR4
MR3
Traits
intense
angry
combative
bossy
aggressive
grumpy
outspoken
condescending
critical
cruel
prejudiced
serious
skeptical
mean
manipulative
defensive
atypical
jealous
self.critical
submissive
helpful
thoughtful
open.minded
weird
happy
optimistic
empathetic
agreeable
patient
leader.like
competent
persistent
wise
ambitious
clever
dignified
well.educated
income
practical
articulate
ethical
courageous
independent
conscientious
punctual
consistent
intellectual
mature
trustworthy
responsible
curious
determined
careful
ignorant
disorderly
unobservant
feminine
anxious
compulsive
sensitive
prudish
emotional
baby.faced
self.pitying
creative
rebellious
confident
strong
energetic
healthy
sociable
flexible
beautiful
charismatic
easygoing
youthful
religious
humble
autistic
traditional
0.00
0.25
0.50
0.75
Loading Strength

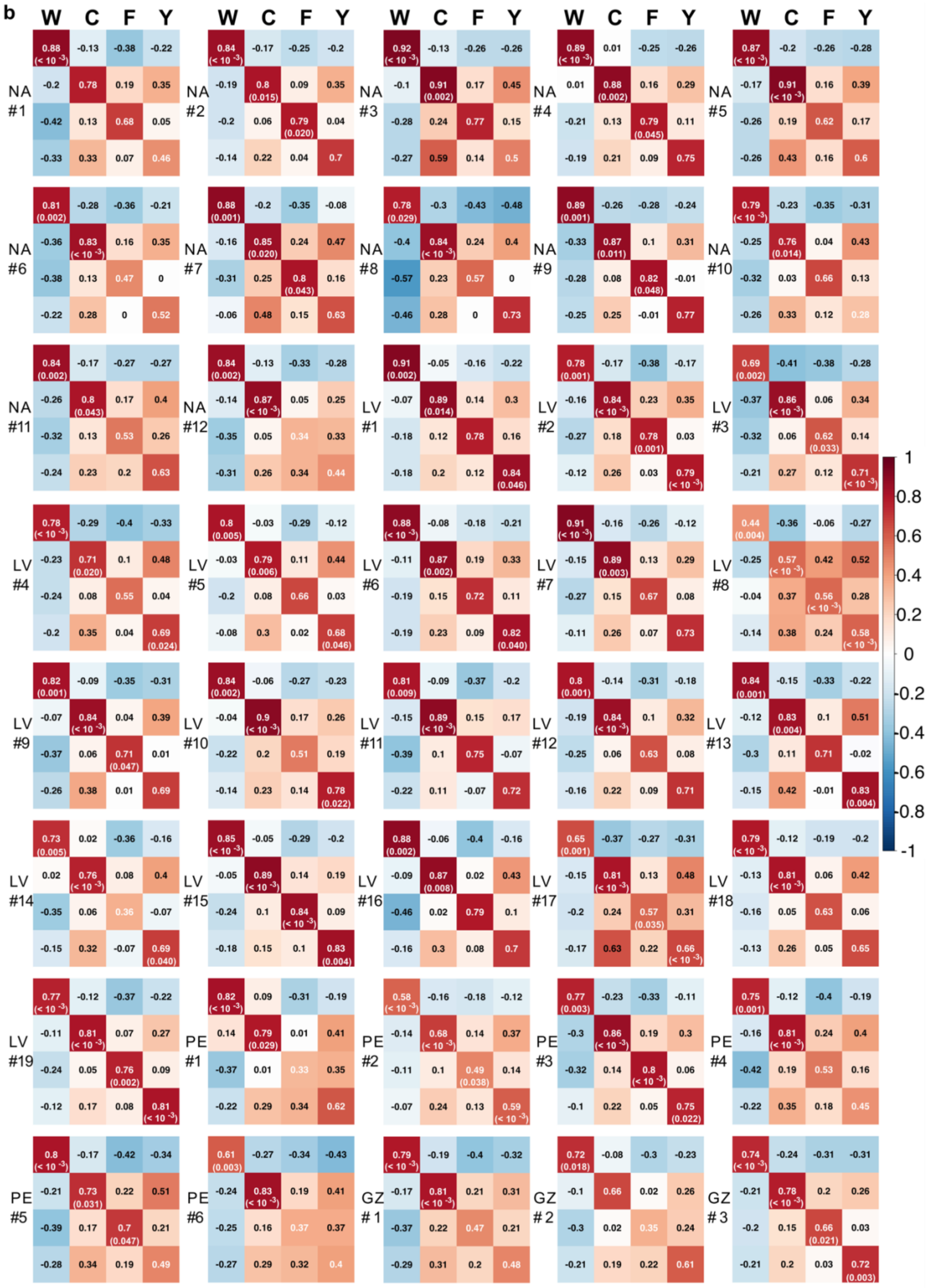
b
W C F Y
NA #1
NA #2
NA #3
NA #4
NA #5
NA #6
NA #7
NA #8
NA #9
NA #10
NA #11
NA #12
LV #1
LV #2
LV #3
LV #4
LV #5
LV #6
LV #7
LV #8
LV #9
LV #10
LV #11
LV #12
LV #13
LV #14
LV #15
LV #16
LV #17
LV #18
LV #19
PE #1
PE #2
PE #3
PE #4
PE #5
PE #6
GZ #1
GZ #2
GZ #3
1
0.8
0.6
0.4
0.2
0
-0.2
-0.4
-0.6
-0.8
-1

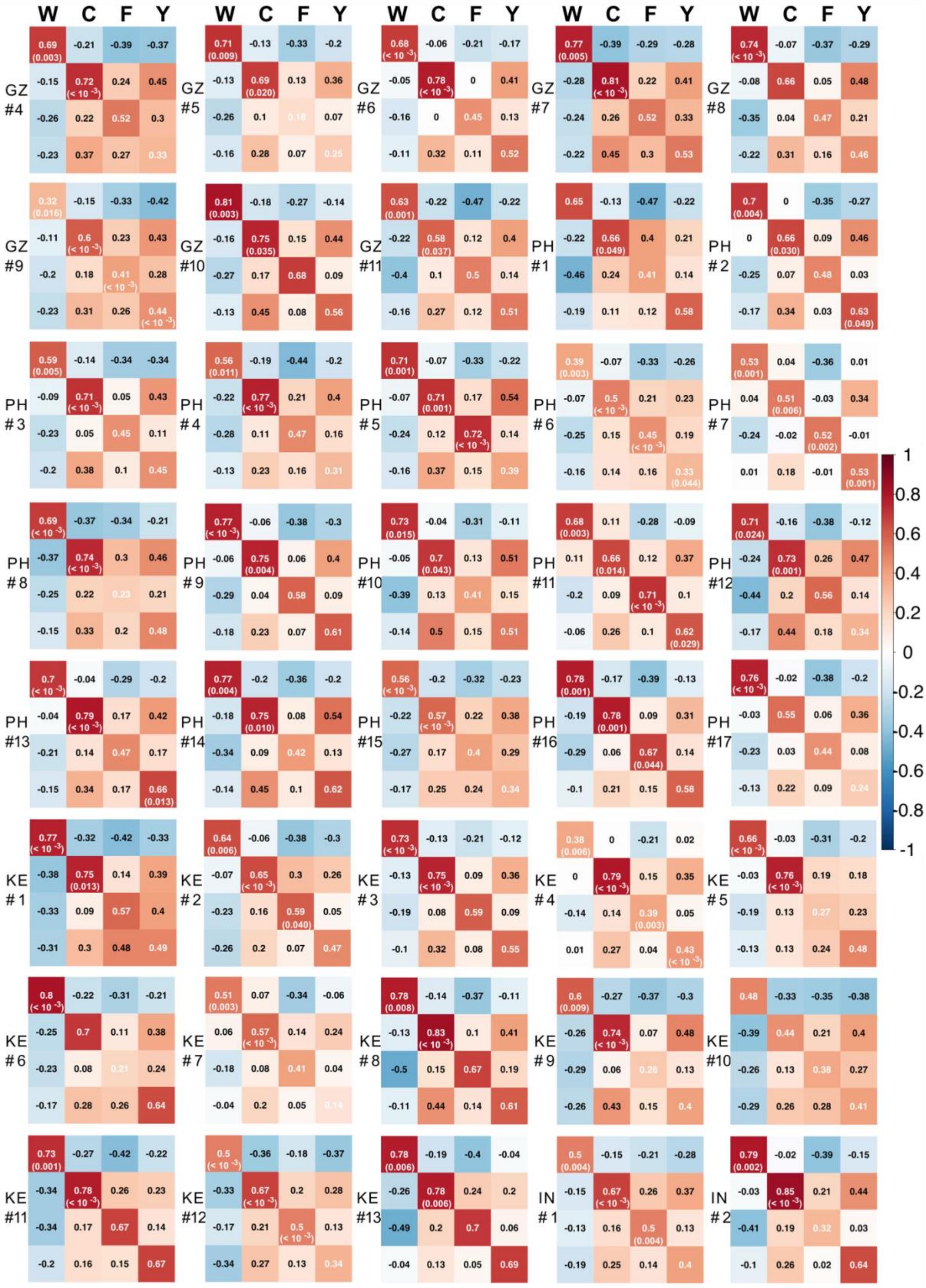
W C F Y
GZ #4
GZ #5
GZ #6
GZ #7
GZ #8
GZ #9
GZ #10
GZ #11
PH #1
PH #2
PH #3
PH #4
PH #5
PH #6
PH #7
PH #8
PH #9
PH #10
PH #11
PH #12
PH #13
PH #14
PH #15
PH #16
PH #17
KE #1
KE #2
KE #3
KE #4
KE #5
KE #6
KE #7
KE #8
KE #9
KE #10
KE #11
KE #12
KE #13
IN #1
IN #2
1 0.8 0.6 0.4 0.2 0 -0.2 -0.4 -0.6 -0.8 -1

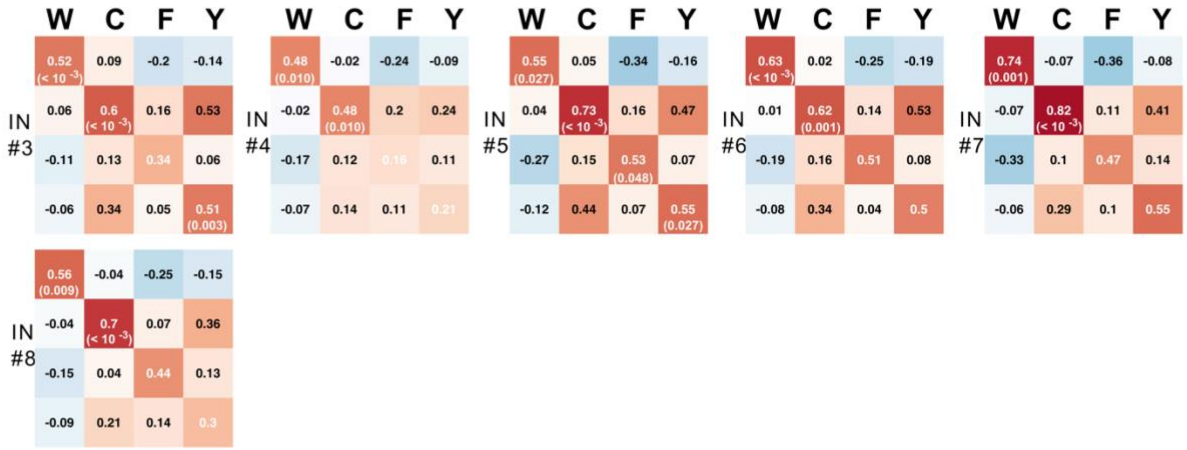

Supplementary Figure 7: Four factors extracted from individual data in Study 2.

a, Examples of the factor loadings from EFA on individual data (a1 North America participant #2, a2 Latvia participant #1, a3 Latvia participant #15). **b,** Factor congruence between Study 1 sample and each individual participant who had a complete dataset in Study 2 (n = 86, who had data for all 80 traits after data exclusion according to preregistered criteria). Each panel plots the Tucker indices of factor congruence (with orthogonal Procrustes rotation) between the four dimensions found in aggregate-level data in Study 1 (columns; W: warmth, C: competence, F: femininity, Y: youth) and those found in individual-level data in Study 2 (rows). The row label of each panel indicates the location (NA for North America, LV for Latvia, PE for Peru, GZ for Gaza, PH for the Philippines, KE for Kenya, IN for India) and the ID of the participant. The numbers report the Tucker indices. The color scale shows the sign and strength of the indices. Statistical significance was obtained using permutation test (with orthogonal Procrustes rotation, and permuting both the rows and columns of the compared factor loading matrix[10] over 1000 iterations); significant p-values ($p < 0.050$) are indicated in parentheses. Source data are provided as a Source Data file.

Supplementary Tables

Supplementary Table 1: Definitions of 100 trait words.

The definition of each trait word was provided to participants in our study to eliminate possible heterogeneity in how each individual understands the meaning of a trait word. These definitions were obtained from Google dictionary, with necessary modifications to make the definition easy to understand and fit the context of describing a person. Additional 6 trait words (idiot, loser, high-income, well-educated, homosexual, autistic) that were not among the popular trait words studied in the literature but also fall under our definition of traits (temporally stable characteristics) were supplemented to the 94 traits selected from the literature. We included these words to be inclusive of traits from the categories of degrading words, demographics and sexual orientations, and mental health conditions.

TRAITS	DEFINITIONS
abusive	A person who is extremely offensive and insulting
affectionate	A person who is comfortable showing his/her love, warmth, and kindness
aggressive	A person who pursues his/her aims and interests forcefully, sometimes with physical force
agreeable	A person who is kind, cooperative, and sympathetic
ambitious	A person who has a strong desire and determination to succeed in their goals
angry	A person who is usually angry
anxious	A person who stresses and worries about things
articulate	A person who speaks fluently and clearly, and who can express their ideas well
atypical	The structure, texture, shape or other aspects of the appearance of the face is unusual or rare
autistic	A person who has autism spectrum disorder—a developmental disorder characterized by troubles with social interaction and communication, and by restricted and repetitive behavior
baby-faced	A person who has facial features resembling a baby
beautiful	A person who looks appealing and physically attractive
bossy	A person who likes giving people orders and wants things his/her own way
careful	A person who works and thinks in a cautious, thorough, or thoughtful way to avoid potential danger
charismatic	A person who is interesting and likeable because they have a charming personality
clever	A person who is quick to understand and learn, and who can figure things out quickly
combative	A person who likes to argue or pick a fight
competent	A person who is efficient and capable to do things in general
compulsive	A person who has to do things in a certain way and often checks and does things over and over again to make sure they are done exactly right
condescending	A person who thinks he/she is better than others and puts other people down
confident	A person who is sure about his/her own abilities, correctness, and successfulness
conscientious	A person who does his/her work or duty thoroughly and responsibly
conservative	A person who sticks to traditional values, especially in politics or religion, and who does not like new ideas or changes
consistent	A person who behaves or responds in the same way over time; reliable
courageous	A person who is not afraid to do the right thing, even if it is dangerous to them
creative	A person who has good imagination or original ideas
criminal	A person who looks like they could commit a crime
critical	A person who judges others harshly, and often makes disapproving comments

cruel	A person who willfully causes pain or suffering to other people or to animals, and feels no concern about it
curious	A person who is eager to learn about or experience new things
defensive	A person who is easily offended and always guards themselves against criticism
determined	A person who is able to make firm decisions and is resolved not to change them
dignified	A person who is polite and composed, and always shows good and respected manners
disorderly	A person who is untidy and not organized
easygoing	A person who is relaxed, tolerant, and not prone to rigid rules or bouts of temper
emotional	A person who shows his/her feelings and laughs and cries easily
empathetic	A person who is able to understand and share the feelings of others
energetic	A person who is very active and full of energy
enthusiastic	A person who is filled with eager enjoyment and interest
ethical	A person who is careful to do things that are morally right to do
feminine	A person whose facial appearance looks like a woman
flexible	A person who is ready and able to change so as to adapt to different circumstances
grumpy	A person who is bad-tempered and always complaining
happy	A person who is usually cheerful
healthy	A person who is in good health
helpful	A person who gives help when others are in need
homosexual	A person who is sexually attracted to people of his/her own sex
humble	A person who is modest and does not boast
idiot	A person who is stupid
ignorant	A person who doesn't know anything, and is also usually unaware of that
income	A person’s income level
independent	A person who is able to think and act without being influenced by others
intellectual	A person who thinks a lot about the deeper meaning of things and likes to analyze things
intense	A person who is very serious and expresses strong feelings
jealous	A person who feels resentment about what other people have
leader-like	A person who can take charge and help a group accomplish a goal
loser	A person who fails frequently or is generally unsuccessful in life
manipulative	A person who likes to control people in order to meet his/her own needs
mature	A person who thinks and behaves like a responsible adult
mean	A person who is unkind, inconsiderate, and doesn't share things
natural	A person who is relaxed and spontaneous
nosey	A person who is overly curious about other people's business
open-minded	A person who is willing to try new things or to hear and consider new ideas
optimistic	A person who is hopeful and confident about the future
outspoken	A person who is frank in stating his/her opinions especially if they are critical or controversial
passive	A person who allows things to happen or accepts what others do, without resistance or trying to change anything
patient	A person who is able to accept or tolerate delays or problems and is very relaxed about getting things done
persistent	A person who is able to continue in a course of action in spite of difficulty or opposition
practical	A person who is sensible and realistic in dealing with a situation or problem
prejudiced	A person who holds biased judgments about other people; bigoted
prudish	A person who is overly proper and cannot stand hearing any sexual reference
punctual	A person who is always on time
reasonable	A person who makes sense and whose opinions most people would agree with
rebellious	A person who resists authority, control, or convention and wants to have their own way
religious	A person who practices religion and believes in their faith
reserved	A person who tends not to show their emotions or opinions and is quiet
responsible	A person who accepts the consequences of his or her own actions and decisions
sarcastic	A person who likes using irony in order to mock others
self-critical	A person who holds himself/herself responsible for any failures, always questioning if they did the right thing or not
self-pitying	A person who feels sorry for themselves
sensitive	A person who is aware of or careful about others' attitudes, feelings, or circumstances
serious	A person who shows deep thoughts and who doesn't smile or laugh easily
shallow	A person who is concerned only about silly or inconsequential things; superficial
sincere	A person who says what he/she genuinely feels or believes
skeptical	A person who questions things and is not easily convinced
sociable	A person who is friendly and enjoys talking and engaging in activities with other people
strict	A person who follows rules exactly, and expects others to follow rules exactly
strong	A person who is physically vigorous and is able to exert great bodily or muscular power

submissive	A person who shows a willingness to be controlled by others or conforms to the authority or will of others
thoughtful	A person who is considerate of others' needs
thrifty	A person who uses money and other resources carefully and not wastefully
traditional	A person who likes to do things the way they have always been done and accepted in the past
trustful	A person who tends to trust other people easily (note: this is different from being trustworthy)
trustworthy	A person who can be relied on as honest and truthful
unobservant	A person who does not notice things
weird	A person who does strange or bizarre things
well-educated	A person who has completed a high level of education, such as bachelor's, master’s and doctorate degrees
white	A person whose face looks like they are Caucasian
wise	A person who has mature experience, knowledge, and good judgments
youthful	A person who looks young

Supplementary Table 2: Robustness check of the four dimensions.

a, Pearson's correlations between our four dimensions (Fig. 3) and the four factors extracted from subsets of traits as a function of their similarity and meaning clarity. All pairs of traits were ranked according to their semantic similarity (using FastText[1], see Methods); for each pair of traits, we removed the one with the lower clarity ratings given by an independent set of participants; we removed traits one by one starting from the pair with the highest similarity to the pairs with lowest similarity. A four-factor solution was extracted from each subset of traits (using the same EFA method as for the full set of traits). To obtain the relations between the factors found in these subsets and those from the full dataset, we assessed the value of every face on each factor (i.e., factor scores, computed using R function factor.scores with method "tenBerge") and then correlated the faces' scores between different factors (two-sided test). b, Factor loadings from EFA on the subset of data corresponding to a smaller subset of specific traits that still yields our four dimensions. The largest absolute loading across four factors for each trait is highlighted in bold. The four dimensions accounted for 88% of the common variance in this subset of data. Source data are provided as a Source Data file.

a

**# traits remaining in the model**	Factor1 and Warmth	Factor2 and Competence	Factor3 and Femininity	Factor4 and Youth	PC1 and Warmth	PC2 and Competence	PC3 and Femininity	PC4 and Youth
91	1.00**	1.00**	1.00**	0.99**	1.00**	1.00**	1.00**	1.00**
90	1.00**	1.00**	0.98**	0.99**	1.00**	1.00**	1.00**	1.00**
89	1.00**	1.00**	0.98**	0.99**	1.00**	1.00**	1.00**	1.00**
88	1.00**	1.00**	0.98**	0.99**	1.00**	1.00**	0.99**	0.99**
87	1.00**	1.00**	0.97**	0.98**	1.00**	1.00**	0.99**	0.99**
86	1.00**	1.00**	0.97**	0.98**	1.00**	1.00**	0.99**	0.99**
85	0.99**	0.99**	0.97**	0.98**	1.00**	1.00**	1.00**	0.99**
84	0.99**	0.99**	0.97**	0.97**	1.00**	1.00**	1.00**	0.99**
83	0.99**	0.99**	0.96**	0.97**	1.00**	1.00**	0.99**	0.99**
82	0.99**	0.99**	0.96**	0.97**	1.00**	1.00**	0.99**	0.99**
81	0.99**	0.99**	0.96**	0.96**	1.00**	1.00**	1.00**	1.00**
80	0.99**	0.99**	0.96**	0.96**	1.00**	1.00**	0.99**	0.99**

79	0.99**	0.99**	0.96**	0.96**	1.00**	1.00**	0.99**	0.99**
78	0.99**	0.99**	0.96**	0.97**	1.00**	1.00**	0.99**	0.99**
77	0.99**	0.99**	0.96**	0.97**	1.00**	1.00**	0.99**	0.99**
76	0.99**	0.99**	0.96**	0.97**	1.00**	1.00**	0.99**	0.99**
75	-0.98**	0.99**	0.95**	0.95**	1.00**	1.00**	1.00**	1.00**
74	-0.98**	0.98**	0.95**	0.94**	1.00**	1.00**	1.00**	1.00**
73	-0.98**	0.98**	0.95**	0.94**	1.00**	1.00**	1.00**	1.00**
72	-0.96**	0.98**	0.95**	0.84**	1.00**	1.00**	1.00**	0.99**
71	-0.96**	0.98**	0.95**	0.83**	1.00**	1.00**	0.99**	0.99**
70	-0.95**	0.98**	0.94**	0.78**	1.00**	1.00**	1.00**	0.99**
69	-0.97**	0.97**	0.95**	0.83**	1.00**	1.00**	1.00**	0.99**
68	-0.97**	0.98**	0.95**	0.83**	1.00**	1.00**	1.00**	0.99**
67	-0.96**	0.98**	0.94**	0.81**	1.00**	1.00**	0.99**	0.99**
66	-0.95**	0.98**	0.94**	0.77**	1.00**	1.00**	0.99**	0.99**
65	-0.94**	0.98**	0.94**	0.72**	1.00**	1.00**	0.99**	0.99**
64	-0.93**	0.98**	0.94**	0.69**	1.00**	1.00**	0.94**	0.94**
63	-0.86**	0.98**	0.93**	0.51**	1.00**	1.00**	0.92**	0.92**
62	-0.85**	0.97**	0.93**	0.49**	1.00**	1.00**	0.93**	0.93**
61	-0.80**	0.98**	0.92**	0.37**	1.00**	1.00**	0.92**	0.93**
60	-0.77**	0.97**	0.92**	0.31*	0.99**	0.99**	0.92**	0.93**
59	-0.76**	0.98**	0.92**	0.28*	0.99**	1.00**	0.94**	0.94**
58	-0.76**	0.98**	0.92**	0.29*	0.99**	1.00**	0.94**	0.94**
57	-0.66**	0.98**	0.93**	0.15	0.99**	0.99**	0.98**	0.97**
56	-0.80**	0.98**	0.92**	0.39**	0.99**	0.99**	0.97**	0.97**
55	-0.98**	0.99**	0.94**	0.91**	0.99**	0.99**	0.98**	0.97**
54	-0.97**	0.98**	0.94**	0.88**	0.99**	0.99**	0.98**	0.97**
53	-0.98**	0.98**	0.94**	0.92**	0.99**	0.99**	0.98**	0.97**
52	-0.82**	0.98**	0.92**	0.39**	0.99**	0.99**	0.99**	0.97**
51	-0.82**	0.98**	0.92**	0.37**	0.99**	0.99**	0.99**	0.97**
50	-0.97**	0.97**	0.94**	0.82**	0.99**	0.99**	0.99**	0.98**
49	-0.97**	0.96**	0.94**	-0.73**	0.99**	0.99**	0.98**	0.98**
48	-0.94**	0.96**	0.93**	0.69**	0.99**	0.98**	0.98**	0.97**
47	-0.95**	0.96**	0.93**	-0.70**	0.98**	0.98**	0.98**	0.97**
46	-0.93**	0.96**	0.92**	0.72**	0.99**	0.98**	0.98**	0.97**
45	-0.96**	0.96**	0.93**	0.76**	0.99**	0.98**	0.98**	0.97**
44	-0.93**	0.95**	0.92**	0.69**	0.99**	0.99**	0.98**	0.97**
43	-0.94**	0.95**	0.92**	-0.66**	0.99**	0.99**	0.98**	0.97**
42	-0.94**	0.94**	0.92**	-0.65**	0.99**	0.99**	0.98**	0.97**
41	-0.94**	0.95**	0.90**	0.67**	0.99**	0.99**	0.97**	0.95**
40	-0.96**	0.94**	0.92**	-0.62**	0.99**	0.98**	0.98**	0.93**
39	-0.96**	0.93**	0.92**	-0.60**	0.99**	0.98**	0.98**	0.93**
38	-0.96**	0.93**	0.92**	-0.55**	0.99**	0.98**	0.98**	0.93**
37	-0.96**	0.92**	0.93**	-0.52**	0.99**	0.98**	0.98**	0.93**
36	-0.96**	0.93**	0.92**	-0.60**	0.98**	0.97**	0.98**	0.93**
35	-0.95**	0.93**	0.92**	-0.62**	0.98**	0.96**	0.98**	0.93**
34	-0.95**	0.93**	0.92**	-0.60**	0.98**	0.96**	0.98**	0.93**
33	-0.94**	0.93**	0.90**	-0.61**	0.98**	0.96**	0.98**	0.91**

32	-0.94**	0.93**	0.88**	-0.63**	0.97**	0.94**	0.97**	0.91**
31	-0.94**	0.93**	0.86**	-0.63**	0.97**	0.94**	0.88**	0.83**
30	-0.95**	0.93**	0.84**	-0.63**	0.96**	0.94**	0.68**	0.62**
29	0.93**	0.90**	0.42**	0.64**	0.96**	0.94**	0.39**	0.26*
28	0.94**	0.89**	0.42**	0.64**	0.97**	0.94**	0.40**	0.29*
27	-0.95**	0.87**	0.40**	0.57**	0.98**	0.95**	0.40**	0.30*
26	-0.95**	0.86**	0.38**	0.66**	0.97**	0.94**	0.38**	0.29*
25	-0.95**	0.85**	0.38**	0.63**	0.98**	0.94**	0.37**	0.25*
24	0.95**	0.87**	0.39**	0.63**	0.99**	0.96**	0.33**	0.20*
23	0.95**	0.82**	0.32*	0.65**	0.99**	0.96**	-0.31*	0.19
22	0.94**	0.89**	0.45**	0.63**	0.99**	0.97**	-0.32*	0.20*
21	0.93**	0.92**	0.49**	0.49**	0.99**	0.97**	0.41**	0.31*
20	0.96**	0.87**	0.38**	0.60**	0.99**	0.97**	0.41**	0.31*

** $p < 0.001$, * $p < 0.05$.

b

Traits	Warmth	Competence	Femininity	Youth
Jealous	**-0.98**	-0.12	0.19	0.18
Critical	**-0.96**	0.18	0.00	0.11
Patient	**0.88**	0.02	0.18	0.08
Easygoing	**0.81**	-0.05	0.05	0.29
Agreeable	**0.75**	0.16	0.10	0.31
Mature	-0.06	**0.99**	0.13	-0.22
Practical	0.16	**0.88**	0.04	0.02
Leader-like	-0.16	**0.83**	-0.11	0.38
Weird	-0.07	**-0.67**	-0.11	-0.34
Idiot	-0.18	**-0.67**	-0.14	-0.34
Strong	-0.11	0.20	**-0.95**	-0.06
Prudish	-0.07	0.17	**0.91**	-0.15
Feminine	-0.07	0.06	**0.91**	0.11
Self-critical	-0.02	0.18	**0.76**	-0.06
Healthy	0.02	0.27	0.02	**0.81**
Youthful	0.11	-0.45	0.23	**0.77**
Charismatic	0.18	0.29	0.09	**0.69**
Sociable	0.22	0.33	0.11	**0.65**

Supplementary References

1. Bojanowski, P., Grave, E., Joulin, A. & Mikolov, T. Enriching Word Vectors with Subword Information. *Transactions of the Association for Computational Linguistics* 135–146 (2017).
2. McInnes, L., Healy, J. & Melville, J. UMAP: Uniform Manifold Approximation and Projection for Dimension Reduction. *arXiv:1802.03426 [cs, stat]* (2018).
3. Bainbridge, W. A., Isola, P. & Oliva, A. The intrinsic memorability of face photographs. *Journal of Experimental Psychology: General* **142**, 1323–1334 (2013).
4. Zhang, Z., Song, Y. & Qi, H. Age Progression/Regression by Conditional Adversarial Autoencoder. in *2017 IEEE Conference on Computer Vision and Pattern Recognition (CVPR)* 4352–4360 (IEEE, 2017). doi:10.1109/CVPR.2017.463.
5. King, D. E. Dlib-ml: A Machine Learning Toolkit. *Journal of Machine Learning Research* 41755–1758 (2009).
6. Ma, D. S., Correll, J. & Wittenbrink, B. The Chicago face database: A free stimulus set of faces and norming data. *Behavior Research Methods* **47**, 1122–1135 (2015).
7. DeBruine, L. & Jones, B. Face Research Lab London Set. (2017) doi:10.6084/m9.figshare.5047666.v3.
8. Velicer, W. F. Determining the number of components from the matrix of partial correlations. *Psychometrika* **41**, 321–327 (1976).
9. Sutherland, C. A. M. *et al.* Social inferences from faces: Ambient images generate a three-dimensional model. *Cognition* **127**, 105–118 (2013).
10. Paunonen, S. V. On Chance and Factor Congruence Following Orthogonal Procrustes Rotation. *Educational and Psychological Measurement* **57**, 33–59 (1997).
