# Supplementary material for: Four dimensions characterize attributions from faces using a representative set of English trait words
Source: Nat Commun. 2021 Aug 27;12:5168. doi: 10.1038/s41467-021-25500-y (PMC8397784; doi:10.1038/s41467-021-25500-y)
Supplement: Supplementary file 3 — Reporting summary [file 41467_2021_25500_MOESM3_ESM.pdf]

nature research

Corresponding author(s): Chujun Lin

Last updated by author(s): Jun 21, 2021

Reporting Summary

Nature Research wishes to improve the reproducibility of the work that we publish. This form provides structure for consistency and transparency in reporting. For further information on Nature Research policies, see our Editorial Policies and the Editorial Policy Checklist.

Statistics

For all statistical analyses, confirm that the following items are present in the figure legend, table legend, main text, or Methods section.

n/a	Confirmed	
☐	☒	The exact sample size (*n*) for each experimental group/condition, given as a discrete number and unit of measurement
☐	☒	A statement on whether measurements were taken from distinct samples or whether the same sample was measured repeatedly
☐	☒	The statistical test(s) used AND whether they are one- or two-sided<br>*Only common tests should be described solely by name; describe more complex techniques in the Methods section.*
☐	☒	A description of all covariates tested
☐	☒	A description of any assumptions or corrections, such as tests of normality and adjustment for multiple comparisons
☐	☒	A full description of the statistical parameters including central tendency (e.g. means) or other basic estimates (e.g. regression coefficient) AND variation (e.g. standard deviation) or associated estimates of uncertainty (e.g. confidence intervals)
☐	☒	For null hypothesis testing, the test statistic (e.g. *F*, *t*, *r*) with confidence intervals, effect sizes, degrees of freedom and *P* value noted<br>*Give P values as exact values whenever suitable.*
☒	☐	For Bayesian analysis, information on the choice of priors and Markov chain Monte Carlo settings
☒	☐	For hierarchical and complex designs, identification of the appropriate level for tests and full reporting of outcomes
☐	☒	Estimates of effect sizes (e.g. Cohen's *d*, Pearson's *r*), indicating how they were calculated

Our web collection on statistics for biologists contains articles on many of the points above.

Software and code

Policy information about availability of computer code

Data collection	Data were collected via experiments using custom codes written in Javascript. Experiment instructions and procedures can be accessed at our preregistrations at Open Science Framework: https://osf.io/6p542/?view_only=fff024253b604edb832a9824cbdafa75 and https://osf.io/qxgmw/?view_only=fd43b2e8b25248f7b7de51b9aeae1894. An example of experiment codes can be accessed at Open Science Framework: https://osf.io/4mvyt/.
Data analysis	Data analysis was performed with R version 3.5.1. and Python version 3.6.9. All analysis codes can be accessed at Open Science Framework: https://osf.io/4mvyt/ and https://osf.io/xeb6w/

For manuscripts utilizing custom algorithms or software that are central to the research but not yet described in published literature, software must be made available to editors and reviewers. We strongly encourage code deposition in a community repository (e.g. GitHub). See the Nature Research guidelines for submitting code & software for further information.

Data

Policy information about availability of data

All manuscripts must include a data availability statement. This statement should provide the following information, where applicable:

- Accession codes, unique identifiers, or web links for publicly available datasets
- A list of figures that have associated raw data
- A description of any restrictions on data availability

The data that support the findings of this study have been deposited in Open Science Framework and can be access at: https://osf.io/4mvyt/ and https://osf.io/xeb6w/. All face images used in this study are from publicly available databases: https://www.chicagofaces.org/ (Chicago Face Database), https://figshare.com/articles/dataset/Face_Research_Lab_London_Set/5047666 (London Face Database), https://sirileknes.com/oslo-face-database/ (Oslo Face Database).

Field-specific reporting

Please select the one below that is the best fit for your research. If you are not sure, read the appropriate sections before making your selection.

☐ Life sciences ☒ Behavioural & social sciences ☐ Ecological, evolutionary & environmental sciences

For a reference copy of the document with all sections, see nature.com/documents/nr-reporting-summary-flat.pdf

Behavioural & social sciences study design

All studies must disclose on these points even when the disclosure is negative.

Study description

Our study is quantitative experimental. We applied deep neural networks to representatively sample multiple stimulus sets, and derived a novel set of 100 traits and 100 faces for a comprehensive protocol we administered in two pre-registered studies. We collected data both on-line and on-site in different countries and regions (North America, Latvia, Peru, the Philippines, India, Kenya, and Gaza). We analyzed the data using linear mixed modeling, exploratory factor analysis, artificial neural network, and representational similarity analysis.

Research sample

The research samples include Amazon Mechanical Turk (MTurk) workers in the United States (Study 1), and participants in North America (Canada and the United States), Latvia, Peru, the Philippines, India, Kenya, and Gaza recruited via the social enterprise Digital Divide Data (Study 2). The MTurk sample (Study 1) is not representative of the U.S. population; any worker with an MTurk account who satisfies the following criteria can participate in our study: located in U.S., HIT approval rate for all requesters' HITs greater than or equal to 95%, aged 18 and older, has normal or corrected-to-normal vision, native English speaker, self-identified as white, the highest level of education has completed is no less than high school (details can be access at our preregistration at Open Science Framework: https://osf.io/6p542/?view_only=fff024253b604edb832a9824cbdafa75). This sample was targeted based on the following rationale: first, an on-line sample was chosen because the experiment procedure allows for an Internet-based data collection, where participants could complete the study at convenient times and places; second, the requirements about HIT approval rate and vision help ensure data quality; third, only participants who self-identified as white were included to match the race of the faces, which in turn help alleviate potential cultural-effects in face perception that we do not intend to investigate in Study 1; lastly, only participants who were located in the U.S., native English speaker, and had completed high school were included to help alleviate noises in participants' understanding of the trait words. The samples in the seven countries and regions (Study 2) are not nationally representative, any participant in the Digital Divide Data subject pools of those locations who satisfies the following criteria can participate in our study: aged 18-40, has been educated and completed at minimum high school, trained in computer skills, proficient in English (except for participants in Peru), has never visited western-cultural countries (except for participants in North America and Latvia); we also targeted an equal ratio of males and females in each location (details can be accessed at our preregistration at Open Science Framework: https://osf.io/qxgmw/?view_only=fd43b2e8b25248f7b7de51b9aeae1894). This sample was targeted based on the following rationale: first, using samples from different parts of the world helps test the generalizability of our results from Study 1; second, using samples from as many different continents as possible and requiring participants in non-western-cultural countries to have minimum cultural exposure to the western cultures allow for a more stringent test of generalizability of our results from Study 1; third, the requirements about education, computer training, and proficient in English help ensure data quality.

Sampling strategy

Participants were randomly sampled from the subject pools satisfying the inclusion criteria stated above. We predetermined sample size based on the point of stability (POS)—a minimum sample size needed to achieve a stable average measure, because most of our analyses used averaged ratings across participants. Our estimation was based on a recent study that analyzed the point of stability for the inferences of 24 traits from faces using 698,829 ratings across 6,593 participants and 3,353 facial stimuli (Hehman, Xie, Ofosu, & Nespoli, 2018). Given that data will be collected on a 7-point Likert scale, the corridor of stability (COS) deemed acceptable to us is +/- 0.5 or +/- 1.00 and the level of confidence deemed acceptable to us is 95%. Across the 24 traits, the POSs ranged from 18 to 42 participants for a COS of +/- 0.5, and from 5 to 11 participants for a COS of +/- 1.00. For the MTurk sample (Study 1), given a large subject pool was available, we predetermined the COS to be +/- 0.5 and the sample size to be 60 participants per trait (details can be access at our preregistration at Open Science Framework: https://osf.io/6p542/?view_only=fff024253b604edb832a9824cbdafa75). For the seven samples in different countries and regions (Study 2), given smaller subject pools were available, we predetermined the COS to be +/- 1.00 and the sample size to be 30 participants per location (details can be accessed at our preregistration at Open Science Framework: https://osf.io/qxgmw/?view_only=fd43b2e8b25248f7b7de51b9aeae1894).

Data collection

Data were collected using computers. The researchers were not presented during any of the data collection procedure. The researchers were not blinded to experimental condition or the study hypothesis. For the Mturk sample (Study 1), workers completed the studies online using their own computers in their own environments. For the seven samples in different countries and regions (Study 2), participants completed the studies on-site using the computers at the local offices of Digital Divide Data.

Timing

MTurk data (Study 1) were collected from April 5, 2018 to July 14, 2018; there was no gap during this data collection period. Cross-cultural data (Study 2) were collected from Dec 10, 2018 to Dec 26, 2018; there was no gap during this data collection period.

Data exclusions

For the MTurk data in Study 1, of the full sample with a registered size of N = 1,500 participants and L = 750,000 ratings, n = 48 participants and l = 27,491 ratings were excluded from further analysis. Data exclusion was performed according to our preregistration: a. Trial-wise deletion would be done if responses are missing or timed out, or if RT is less than 100ms; b. Participant-wise deletion would be done if a participant has more than 10% of invalid trials in any block as per (a); c. Block-wise (trait-wise per subject) deletion would be done if all trials in a given block have the same rating (detail can be accessed at https://osf.io/6p542/?view_only=fff024253b604edb832a9824cbdafa75). For the cross-cultural data in Study 2, we have preregistered data exclusion criteria in our initial preregistration (https://osf.io/qxgmw/?view_only=fd43b2e8b25248f7b7de51b9aeae1894) and our second preregistration (https://osf.io/tbmsy/?view_only=6d8b94575bf0469fb157c89eb9292371). According to the preregistered data

exclusion criteria A to C (for the reliability and consensus analyses), of the full sample with a preregistered size of N = 30 participants and L = 300,000 ratings at each of 7 locations (N = 210 total), we excluded from further analysis n = 1 participant in India and l = 24,236 ratings in North America, l = 2,507 ratings in Latvia, l = 16,366 ratings in Peru, l = 3,178 ratings in the Philippines, l = 14,389 ratings in India, l = 9,117 ratings in Kenya, and l = 4,096 ratings in Gaza. According to the preregistered data exclusion criteria A to D (for the dimensional analysis with aggregate-level data), 31 participants across seven locations were excluded for further analysis (n = 3 for North America, n = 2 for Latvia, n = 7 for Peru , n = 3 for the Philippines, n = 10 for India, n = 2 for Kenya, and n = 4 for Gaza). For the dimensional analysis with individual-level data, only participants who had complete trait-wise data after exclusion criteria A to D were used in the analysis (n = 86).

Non-participation	For the Mturk sample (Study 1), no participants dropped out/declined participation. For the cross-cultural samples (Study 2), no participants dropped out/declined participation in North America and Latvia; one participant dropped out in Gaza; two participants dropped out in Kenya; two participants dropped out in India; four participants dropped out in the Philippines; eight participants dropped out in Peru. These participants dropped out because they were no longer able to commit to completing all 20 experiment modules in ten business days. All dropped-out participants have been replaced with new participants so that the final sample size met the preregistered sample size (N = 30 in each location).
Randomization	For the Mturk sample (Study 1), participants were randomly allocated to one of the 25 modules. For the cross-cultural samples, a complete dataset was collected from each participant so participants were not allocated into experimental groups.

Reporting for specific materials, systems and methods

We require information from authors about some types of materials, experimental systems and methods used in many studies. Here, indicate whether each material, system or method listed is relevant to your study. If you are not sure if a list item applies to your research, read the appropriate section before selecting a response.

Materials & experimental systems

n/a	Involved in the study	
☒	☐	Antibodies
☒	☐	Eukaryotic cell lines
☒	☐	Palaeontology and archaeology
☒	☐	Animals and other organisms
☐	☒	Human research participants
☒	☐	Clinical data
☒	☐	Dual use research of concern

Methods

n/a	Involved in the study	
☒	☐	ChIP-seq
☒	☐	Flow cytometry
☒	☐	MRI-based neuroimaging

Human research participants

Policy information about studies involving human research participants

Population characteristics	For the Mturk sample (Study 1): N = 1,500 (800 males), Age(M = 38 years, SD = 11), median of educational attainment was “some post-high-school, no bachelor's degree”. All participants were required to be white, native English speakers, located in the U.S., and 18 years old or older. We also collected data about whether our participants were currently being treated for psychiatric or neurological illness. The majority of our participants (79.7%) were not currently being treated for any psychiatric or neurological illness. The rest were currently being treated for depression (9.8%), bipolar disorder (1.3%), anxiety or panic disorder (11.2%), obsessive compulsive disorder (0.9%), post-traumatic stress disorder (1.3%), autism spectrum disorder (0.3%), learning disability (0.1%), attention deficit (0.9%), alcohol or drug addiction (1.0%), personality disorder (0.5%), dissociative disorder (0.1%), epilepsy (0.2%), and brain injury (0.1%). For the cross-cultural samples (Study 2), N = 30 participants per location (15 females and 15 males) were recruited as planned. Age (M = 26, SD = 4) for North America; Age (M = 22, SD = 3) for Peru; Age (M = 28, SD = 5) for Latvia; Age (M = 26, SD = 5) for Gaza; Age (M = 24, SD = 2) for Kenya; Age (M = 27, SD = 6) for India; Age (M = 25, SD = 4) for Philippines.
Recruitment	For Study 1, participants were recruited via Mturk; any worker who satisfies our preregistered inclusion criteria (see above, Research sample) can participate in our study. For Study 2, participants were recruited via Digital Divide Data, a social enterprise that delivers research services, in seven countries/regions of the word: North America (U.S. and Canada), Latvia, Peru, the Philippines, India, Kenya, and Gaza. Any worker in their subject pools of the seven locations who satisfies our preregistered inclusion criteria (see above, Research sample) can participate in our study. One potential self-selection bias might be that individuals who are more interested in our study are more likely to participate. This is not likely to impact the results but these individuals are likely to have paid more attention during the study and therefore might have provided data with higher quality (e.g., higher test-retest reliability, fewer missing trials).
Ethics oversight	Institutional Review Board of California Institute of Technology

Note that full information on the approval of the study protocol must also be provided in the manuscript.
